# Mechanistic Understanding of Lithium-Ion Adsorption, Intercalation, and Plating during Charging of Graphite Electrodes

**DOI:** 10.1021/acselectrochem.4c00079

**Published:** 2025-04-22

**Authors:** Brian Chen, Niya Hope-Glenn, Amanda Wright, Robert J. Messinger, Alexander Couzis

**Affiliations:** Department of Chemical Engineering, The City College of New York (CUNY), 160 Convent Avenue, New York, New York 10031, United States

**Keywords:** Lithium-Ion Batteries, Electrochemical Reaction Mechanisms, Langmuir Adsorption, Low-Temperature Charging, Fast-Rate Charging

## Abstract

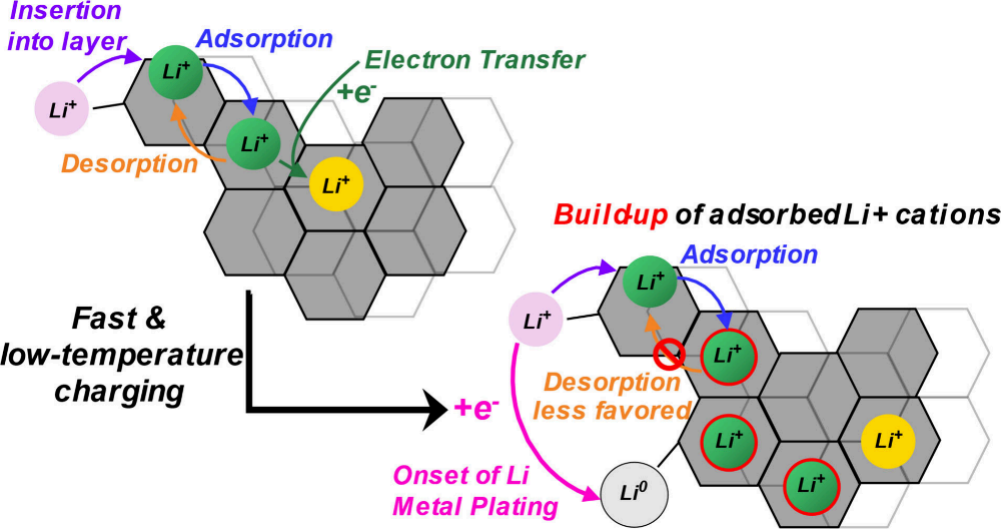

Low-temperature and fast-charging lithium (Li)-ion batteries remain challenging due to the undesirable Li plating on graphite anodes under these conditions. Here, we present a kinetic mechanism that underpins electrochemical Li^+^ cation intercalation and Li metal plating reactions on graphite electrodes at low temperatures and fast rates. Variable-temperature (30 °C to −40 °C) and variable-rate (0.1 to 10 mA/cm^2^) constant-current measurements were conducted on three-electrode cells comprised of Li metal counter, graphite working, and Li metal reference electrodes, as well as two-electrode cells. The local minima in the potential profiles, often associated with the nucleation overpotential for Li metal plating on graphite, must be disentangled from contributions from Li metal stripping at the counter electrode. Differential capacity analyses of three-electrode measurements of graphite potential show that the extent of electrochemical Li^+^ cation intercalation drops precipitously as temperature decreases below −20 °C. The temperature dependence of empirically defined rate constants for Li^+^ cation intercalation and Li plating determined from constant-current measurements revealed non-Arrhenius behavior for Li^+^ cation intercalation that suggests a two-step pre-equilibration mechanism, while typical Arrhenius behavior for Li plating suggests a unimolecular single-step process. A kinetic model based on Langmuir adsorption shows that the interfacial concentration of Li^+^ cations adsorbed on graphite active sites is critical in dictating the kinetics of the charging process. We show that rate limitations, either adsorption-limited or surface reaction-limited, manifest at different temperatures and rates during the charging process. The results yield new mechanistic understanding of how Li^+^ cations electrochemically compete for intercalation into and plating on graphite electrodes, as a function of temperature and charge rate.

## Introduction

Many technologies use Li-ion batteries to execute complex functions. Li-ion batteries have two significant limitations that must be resolved to improve their function: (i) they are not able to be charged within the desired 10-15 min timeframe, a disadvantage compared to gas-powered vehicles,^1−3^ and (ii) do not function well at low temperatures (<−20 °C)^4,5^ or high temperatures (>60 °C),^6,7^ which can be required for demanding applications ranging from terrestrial electric vehicles charging in colder climates^8^ to robotic spacecraft exploring the harsh surfaces of extraterrestrial planets.^6^

Li-ion battery rechargeability depends on the reversible electrochemical intercalation of Li^+^ cations in both the anode (e.g., graphite) during charging and in the cathode (e.g., transition metal oxides like LiNi_0.8_Mn_0.1_Co_0.1_O_2_) during discharging. However, Li electrodeposition (i.e., Li plating) can occur instead of Li^+^ cation intercalation on graphite electrodes during the charging process, especially at low temperatures and fast rates.^9^ Li plating involves electrochemically reducing Li^+^ cations from the electrolyte to form Li metal on the surface of graphite anodes. Once plated on the graphite surface, Li metal can accelerate cell failure by reacting with the electrolyte further to form a solid electrolyte interphase (SEI) layer,^10^ dendrites that could short-circuit the cell and lead to thermal runaway,^11^ decrease the available capacity of the Li-ion battery through formation of Li that becomes electrochemically inactive (so-called “dead lithium”),^12^ and block active sites on graphite that prevents Li^+^ cation intercalation. Thus, the issue of Li plating must be addressed to expand the use of Li-ion batteries to new and challenging applications.

Researchers have established that Li plating occurs when the graphite electrode potential reaches 0 V vs Li/Li^+^ and below.^10,13−16^ Fast-rate and low-temperature charging polarizes the graphite electrode to potentials below 0 V vs Li/Li^+^ due in part to the sluggish kinetics of Li^+^ cation intercalation,^17,18^ which produces an overpotential that drives the graphite potential towards 0 V and below. Therefore, strategies to mitigate Li plating on graphite have generally centered on improving the rate of Li^+^ cation intercalation. Such strategies have included: (i) using chemical/physical graphite surface treatments to improve the accessibility of active sites for Li^+^ cation intercalation,^19−21^ and (ii) formulating new electrolytes that decreases the Li^+^ de-solvation energy barrier.^4,5,22−26^ It is well-known that de-solvation is a rate-limitation at colder temperatures.^27−29^

Despite advances in improving the kinetics of Li^+^ cation intercalation for fast-rate and low-temperature charging, the Li nucleation process on graphite electrodes under galvanostatic conditions remains unclear. Mechanistic studies of metal nucleation typically involve constant-potential measurements,^30^ partly because it is straightforward to experimentally establish the thermodynamic driving force for electrodeposition. For example, Verbrugge and Koch^16^ used potential holds to establish that graphite electrodes can tolerate potentials below 0 V vs. Li/Li^+^ for some time before Li plating. Constant-potential experiments at −50 mV, −100 mV, and −200 mV v. Li/Li^+^ showed that Li^+^ cations can intercalate at potentials negative to the Li metal equilibrium (0 V vs Li/Li^+^) followed by Li plating (e.g., within 400 s at −200 mV). These results indicate that a critical transition from electrochemical Li^+^ cation intercalation to Li plating on graphite can occur even at potentials below 0 V vs. Li/Li^+^, where both intercalation and plating are far from equilibrium.

The onset of Li plating is often determined electrochemically by measurement of the “nucleation overpotential” (*η*_*nuc*_) in a two-electrode cell configuration under constant-current conditions. The nucleation overpotential represents the energy barrier required to nucleate and stabilize the initial deposition of metal on an electrode surface, and its measurement has been reported to appear as a local minimum at cell potentials below 0 V vs a metal reference electrode.^31,32^ Thus, for graphite electrodes charging under a constant-current, the graphite potential can remain negative for some time prior to reaching *η*_*nuc*_. The molecular-level events that underpin the metal nucleation process leading up to and at *η*_*nuc*_ are unclear. For instance, Yuan et al.^33^ reported that the electrode potential leading to *η*_*nuc*_ can be attributed to electric double-layer charging for gold electrodeposition. Ely and Garcia,^34^ however, developed a model for electrochemical heterogeneous nucleation that suggests that the electrode potential leading to *η*_*nuc*_ points to the simultaneous formation and dissolution of neutral, unstable metal embryos on the electrode surface.

Classical nucleation theory is typically invoked to relate the size of electroplated metal particles to measurements of *η*_*nuc*_ under constant-current conditions.^34−37^ The critical radius size (*r*_*crit*_) for the nucleating metal is inversely proportional to *η*_*nuc*_ by the following relation (eq 1):
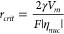
1where *γ* is the interfacial tension, *V*_*m*_ is the molar volume of the depositing metal, and *F* is Faraday’s constant. Increases in charging current and/or decreasing the temperature result in an increase in *η*_*nuc*_ (due to polarization) and a decrease in *r*_*crit*_. The nucleation overpotential *η*_*nuc*_, and consequently *r*_*crit*_, affects the resulting particle size during growth.^38^ For instance, reports have shown that high *η*_*nuc*_ results in the formation of fine metallic deposits, evidenced by electron microscopy images of Li^35−37^ and zinc^39^ metal plated on various working electrodes.

Herein, we investigated the kinetics of electrochemical Li^+^ cation intercalation and Li plating reactions on graphite using empirically defined rate constants for both reactions obtained under constant-current. The temperature dependence of these rate constants for Li^+^ cation intercalation and Li plating points to a mechanism where Li^+^ cations adsorb on the graphite surface before either Li^+^ intercalation or Li plating occurs. Temperature and/or charging current can affect the kinetics of the Li^+^ cation adsorption process and, thus, the interfacial concentration of adsorbed Li^+^ cations available for electrochemical ion intercalation or plating reactions. Specifically, we forced Li to plate on graphite under a constant-current in both two- and three-electrode Li/graphite cell configurations and temperatures from 30 °C down to −40 °C and at different current densities from 0.1 mA/cm^2^ to 10 mA/cm^2^.

## Experimental Methods

### Two- and Three-Electrode Electrochemical Cells

**Figure 1:**
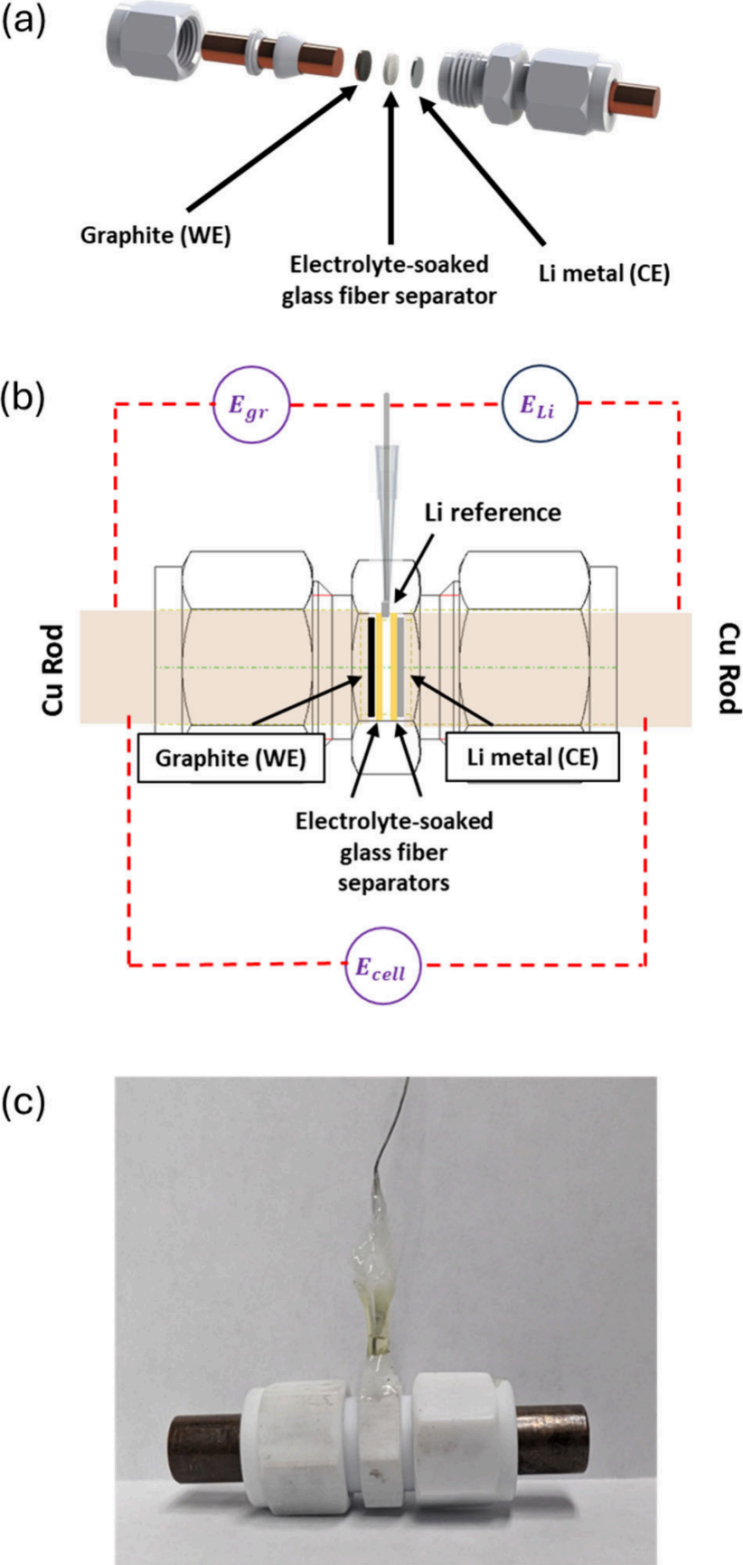
Schematics of (a) two-electrode Li/graphite cell, (b) three-electrode Li/Li/graphite cell showing the notation for measurements of the graphite (*E*_*gr*_), Li metal (*E*_*Li*_), and full-cell (*E*_*cell*_) potential. (c) Image of the three-electrode cell configuration used for constant-current measurements.

A two-electrode, -inch” polytetrafluoroethylene (PTFE) union Swagelok cell was used for constant-current Li plating measurements (Figure 1a). The counter electrode/reference electrode was Li metal foil (battery-grade, 200 μm, China Energy Lithium Co., LTD), and the working electrode was graphite (NEI Corp., Nanomyte BE-200E; theoretical capacity = 372 mAh/g). Glass fiber (Whatman GF/D) separators were used. Graphite electrodes have a mass loading of 6.5 mg/cm^2^ ± 5%, an areal loading of 2.4 mAh/cm^2^ ± 5%, and a specific surface area of 1.5–1.8 m^2^/g. 30 μL of 1 M LiPF_6_ in EC/DMC/DEC (1:1:1 (v/v)) (Sigma-Aldrich, 9016856) was used. All two-electrode Li/graphite cells were constructed in an argon-filled glovebox with <1 ppm H_2_O and <1 ppm O_2_. The surrounding cell temperature was controlled in a convection oven (ESPEC BTZ-133 or Arbin multi-zone temperature chamber, both ±0.5 °C). Temperature equilibration was assumed to be reached in 1–2 h. All electrochemical experiments were done without any iR compensation. The open-circuit potential of all Li-metal, two- and three-electrode cells was ∼3.0 V to 3.2 V.

Three-electrode Li/Li/graphite cells were constructed to de-couple the Li metal counter electrode (*E*_*Li*_) and the graphite working electrode (*E*_*gr*_) potentials from the composite Li/graphite response (*E*_*cell*_). Both *E*_*Li*_ and *E*_*gr*_ are referenced vs a Li metal reference electrode, while *E*_*cell*_ represents the electrode potential difference *E*_*cell*_ = *E*_*gr*_ – *E*_*Li*_ (Figure 1b). A 2 mm hole was drilled in the center of a PTFE Swagelok union (0.5 in. diameter) to accommodate the wire reference electrode and threaded with a 3 mm × 0.5 mm tap. The Li metal reference electrode was prepared in the glovebox by wrapping a small piece of Li metal around the tip of the nickel (Ni) wire (ThermoScientific; 0.5 mm; 99.5%). The Li reference electrode (with Ni wire) was then inserted through a pipette tip through the center of the PTFE union. Two glass fiber separators were used to isolate the inserted Li reference electrode. 270-300 μL of electrolyte (1 M LiPF_6_ in EC/DMC/DEC (1:1:1 (v/v))) was added to the cell. Epoxy (Devcon) was used to seal the connection between the Li reference electrode and the Swagelok union (Figure 1c).

### Constant-Current Measurements of Li^+^ Intercalation and Li Plating on Graphite

All constant-current experiments for two-electrode Li/graphite cells were conducted using a 64-channel battery cycler (Arbin Instruments LBT). A potentiostat (BioLogic VSP-300) was used for three-electrode Li/Li/graphite cells. Because Li metal was used as the counter electrode (i.e., anode) for all measurements, the electrochemical behavior of graphite (i.e., cathode) was measured under a “discharging” current, resulting in a decrease in potential. Herein, the measured voltage profile of the graphite electrode in the three-electrode configuration will be referred to as a “charging process” to stay consistent with graphite acting as an anode during charge in a full-cell, two-electrode Li-ion battery using a transition metal oxide (e.g., LiNi_0.8_Mn_0.1_Co_0.1_O_2_ (NMC811)) as a cathode. In either discharging a Li/graphite cell or charging a graphite/NMC811 Li-ion battery, Li^+^ cations are transported to the graphite electrode, resulting in either intercalation or plating. All assembled cells were allowed to rest for 1 h at temperatures between −40 °C and 30 °C before the constant-current density step between 0.1 mA/cm^2^ and 10 mA/cm^2^. The corresponding C-rates for the current densities used in this study are approximately C/24 (0.1 mA/cm^2^) to 4.13C (10 mA/cm^2^), where C was defined with respect to the theoretical specific capacity of graphite (372 mAh/g) and calculated based on the active material loading (6.5 mg/cm^2^).

Two- and three-electrode cells were either (i) charged as is after temperature equilibration without formation cycling or (ii) charged after subjecting the graphite electrode to formation cycling. Specifically, to assess the effect of the SEI layer on the Li^+^ cation intercalation and Li plating processes, two-electrode -inch” PTFE Swagelok cells comprised of NMC811 (NEI Corp., Nanomyte BE-56E; theoretical capacity = 200 mAh/g) as cathode and graphite as anode underwent formation cycling at room temperature to build the SEI layer on graphite. Aluminum and copper rods were used as current collectors for NMC811 and graphite electrodes, respectively. Glass fiber (Whatman GF/D) was used as separator with 120 μL of 1 M LiPF_6_ in EC/DMC/DEC (1:1:1 (v/v/v)).

Formation cycling for SEI studies consisted of a C/20 constant-current, constant-voltage charge to 4.2 V with a C/50 current taper followed by a 15 min rest after charge. After the 15 min rest, a C/20 discharge to 2.5 V was conducted followed by another 15 min rest (Figure S1, Supporting Information). This formation protocol was repeated three times. C-rates for formation cycling were based on the theoretical specific capacity of graphite. After formation cycling, NMC811/graphite cells were disassembled in an argon-filled glovebox where the graphite electrode was harvested and rinsed with dimethyl carbonate (ThermoScientific, 99+%, Extra Dry, AcroSeal), and dried under vacuum overnight. Dried graphite electrodes were then placed in three-electrode Li/Li/graphite cells (Figure 1b,c) with fresh glass fiber separators and electrolyte, and charged at 0.1 mA/cm^2^ at temperatures between 20 °C and −40 °C.

Differential capacity analysis (d*q*/d*V*) on all constant-current measurements was conducted using a moving average (Text S1, Figure S2, and Figure S3, Supporting Information). This analysis provides information on redox reactions occurring during either charge or discharge. For Li^+^ cation intercalation into graphite measured in a half-cell configuration where Li metal is the anode, the typical d*q*/d*V* possesses four peaks indicative of the graphite staging transitions (Figure S4, Supporting Information). For 0.1, 1, 5, and 10 mA/cm^2^, d*q*/d*V* plots were calculated using 200, 20, 4, and 2 data points for the moving average, respectively.

## Results and Discussion

### Li^+^ Intercalation and Li Plating in Two- and Three-Electrode Cell Configurations

**Figure 2:**
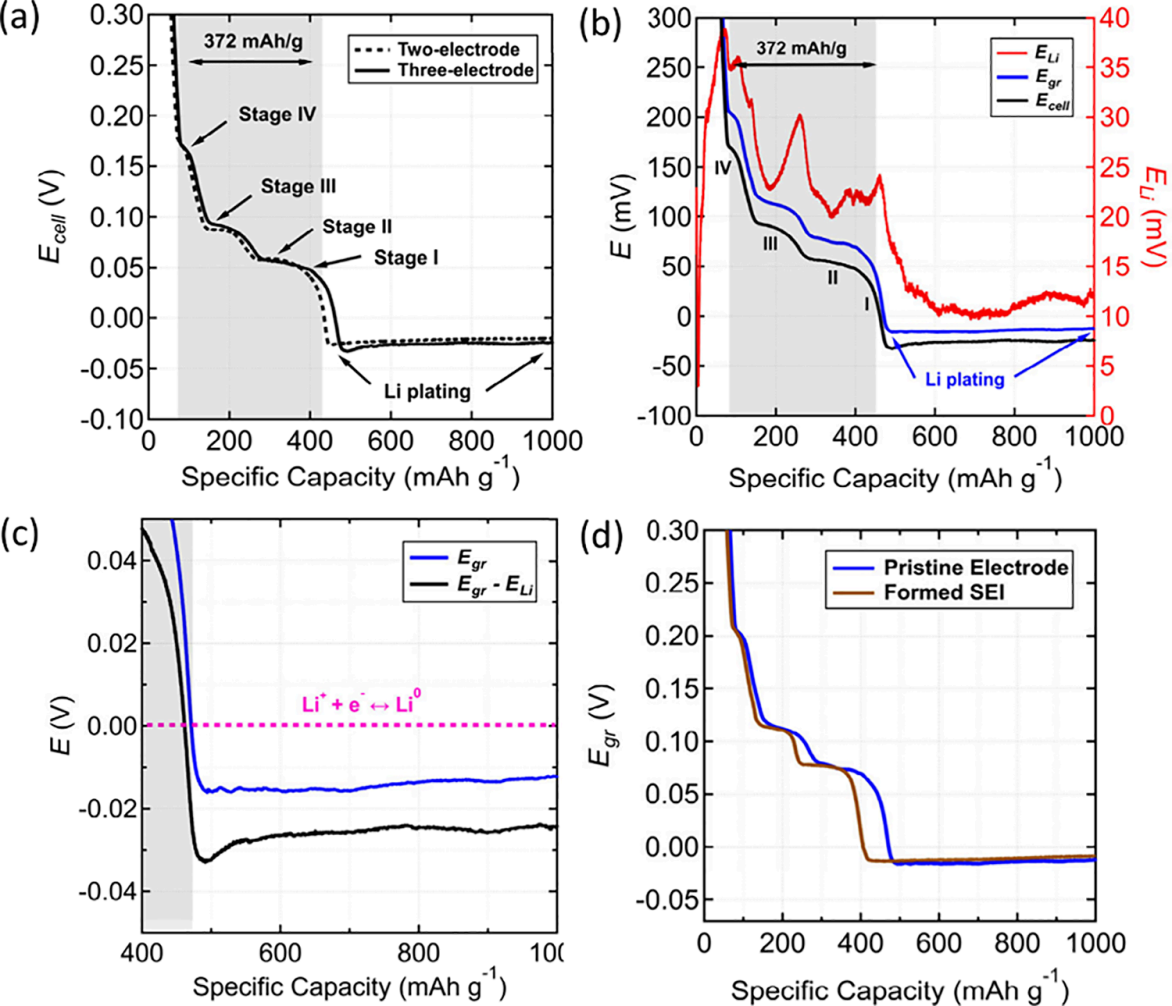
Constant-current measurements of two- and three-electrode Li/graphite cells at baseline conditions of 20 °C and 0.1 mA/cm^2^. (a) Cell potential profile of two-electrode Li/graphite cell (dashed black) in comparison to three-electrode full-cell (solid black) measured without formation cycling. Graphite staging and onset of Li plating are labeled. Graphite’s theoretical capacity (372 mAh/g) is highlighted and coincides with the stage 4 to stage 1 transition. (b) Electrode potential profiles in a three-electrode cell configuration measured without formation cycling. The graphite electrode potential (solid blue, *E*_*gr*_), the Li metal electrode potential (solid red, *E*_*Li*_), and the Li/graphite full-cell potential (solid black, *E*_*cell*_) are shown, where the graphite staging and Li plating are labeled. The *E*_*cell*_ that was obtained in the three-electrode cell is identical to the two-electrode response in panel a. (c) Zoomed in *E*_*gr*_ and *E*_*cell*_ profiles from panel b for the three-electrode cell at potentials below 0 V. The Li equilibrium redox is shown (dashed pink). (d) Three-electrode *E*_*gr*_ profile without formation cycling (identical to *E*_*gr*_ in panel b) and another three-electrode *E*_*gr*_ profile in which the graphite electrode underwent prior formation cycling in a two-electrode NMC811/graphite cell.

We first established the typical electrode potential profile of graphite during the charging process in two- and three-electrode cells (Figure 2). The constant-current response of graphite was measured in a two-electrode Li/graphite and a three-electrode Li/Li/graphite cell configuration using Li metal as the counter (CE)/reference electrode and graphite as the working electrode (WE). During charge in a two- and three-electrode cell, the cell potential profiles first encounter shoulders at ∼0.7 V (Figure S5, Supporting Information), which is attributed to electrolyte reduction for forming a primitive SEI-like layer on graphite.^40^ Upon further charge, graphite staging can be observed up to stage 1, which corresponds to the full theoretical capacity of graphite (372 mAh/g).^40^

Beyond the attainment of 372 mAh/g, the cell potential continues to decrease at around 400 mAh/g below 0 V, where the nucleation overpotential *η*_*nuc*_ is reached. Beyond the local minimum, the cell potential rises steadily. Gao et al.^17^ reported optical microscopy images to confirm the presence of Li plating beyond the cell potential minimum on highly oriented pyrolytic graphite electrodes. The steady rise in cell potential beyond the minimum can be understood as the electrodeposition of Li on Li metal.^35,38^ Significant work has been done on understanding the morphological transitions of Li metal during its growth process. For example, reports have established that Li metal transitions from a mossy to a dendritic morphology at the Sand’s time, indicating a switch from kinetic-controlled growth to diffusion-controlled growth.^42−44^ Dendrite formation for Li electrodeposition is typically studied in the context of Li metal anodes for Li metal batteries.^45−47^

Interestingly, the three-electrode measurement (Figure 2b) revealed that the cell potential minimum in *E*_*cell*_ arises because of the overpotential for electrostripping the Li metal CE (Figure 2c), which caused a ∼15 mV difference between *E*_*cell*_ and *E*_*gr*_. Instead, the *E*_*gr*_ profile assumed a plateau up to 700 mAh/g followed by a steady rise. Cai et al.^48^ reported scanning electron micrographs that showed the appearance of Li metal on the surface of graphite electrodes plated beyond the nucleation overpotential, and can be explained as a transition from Li plating within the pores of the graphite electrode to plating on the graphite electrode surface. A three-electrode cell is required for measuring the graphite electrode response and quantitatively distinguishing it from the Li metal CE. This is recommended by prior works encouraging the use of a Li metal reference electrode^32,49^ and aids in the mechanistic understanding of graphite electrode processes.

Graphite electrodes formed in two-electrode NMC811/graphite cells were harvested and placed into a three-electrode Li/Li/graphite cell to study how a pre-formed SEI affects the electrochemical Li^+^ cation intercalation and Li plating constant-current measurements. The graphite potential *E*_*gr*_ was measured and compared to otherwise identical cells without a pre-formed SEI (Figure 2d). Under the same conditions, the measured graphite potential *E*_*gr*_ shows qualitatively similar behavior. The plateaus associated with graphite staging and the Li plating minimum were observed at identical potentials, demonstrating that a SEI layer has a negligible effect on the constant-current measurements of graphite. This result suggests that Li^+^ cation reduction on graphite during charge is independent of the presence and the chemical composition of the SEI.

### Variable-Temperature Three-Electrode Measurements of Graphite

The three-electrode measurement of *E*_*gr*_ and *E*_*cell*_ at 20 °C and 0.1 mA/cm^2^ (Figure 2c) is a baseline measurement where the graphite electrode achieves lithiation to LiC_6_ at a relatively slow charge current (0.1 mA/cm^2^ corresponding to ∼C/24 rate) prior to Li plating. At these conditions, forming a SEI layer prior to the constant-current measurement has a negligible effect on the graphite potential *E*_*gr*_ during Li^+^ cation intercalation and Li plating.

**Figure 3:**
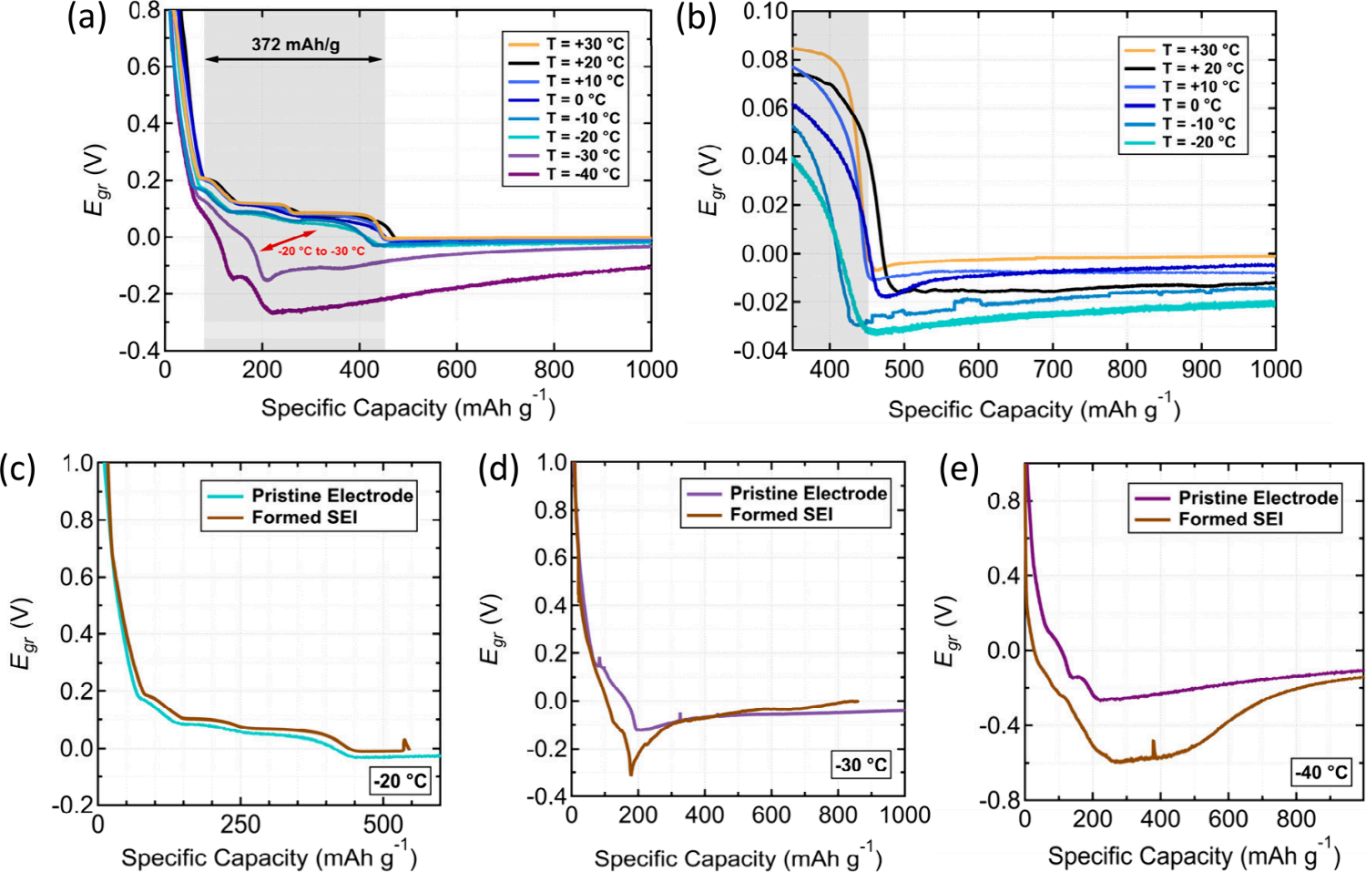
(a) Variable-temperature, constant-current measurements conducted in a three-electrode Li/Li/graphite cell at 0.1 mA/cm^2^ from 30 °C to −40 °C without prior formation cycling. (b) Expanded data range from panel a to show the onset of Li plating on graphite at temperatures between 30 °C and −20 °C. The effect of prior formation cycling on the *E*_*gr*_ measurement is shown at (c) −20 °C, (d) −30 °C, and (e) −40 °C.

Variable-temperature three-electrode measurements were conducted at a constant current of 0.1 mA/cm^2^ and in a temperature range from 30 °C down to −40 ° C to understand the effects of temperature on *E*_*gr*_ (Figure 3a). Experimenting with temperature provides insight into the mechanism of the Li^+^ cation reduction process on graphite. In a three-electrode measurement where *E*_*gr*_ and *E*_*Li*_ were measured against a Li metal reference electrode, the electrode potential difference *E*_*cell*_ between the graphite WE and Li metal CE matches the two-electrode Li/graphite cell potential profiles over all of the temperatures tested (Figure S6, Supporting Information). This result demonstrates experimental consistency in measuring the electrochemical behavior of Li/graphite charging for both cell designs across the entire temperature range. Measurements of *E*_*cell*_ and *E*_*Li*_ showed that the overpotential for stripping the Li metal CE increases as the temperature decreases (Figure S7, Supporting Information).

The three-electrode graphite potential measurements at temperatures between 30 °C and −20 °C (Figure 3b) were similar to the baseline measurement at 20 °C (Figure 2b), where the plateaus associated with graphite staging were well-defined and contributed to the near theoretical capacity of graphite. However, as the temperature decreased from −20 °C to −30 °C or −40 °C, a rapid decrease in the graphite potential *E*_*gr*_ was observed. At −30 °C and −40 °C, *E*_*gr*_ crossed 0 V vs Li/Li^+^ at 160 mAh/g and 106 mAh/g, respectively, compared to 412 mAh/g at −20 °C. This earlier approach to 0 V vs Li/Li^+^ indicates an inability to achieve the full intercalation capacity at colder temperatures.

Interestingly, this abrupt transition between −20 °C and −30 °C to −40 °C was observed when intercalating ions other than Li^+^ cations. For example, in our prior work studying chloroaluminate anion (AlCl_4_^-^) intercalation in graphite for aluminum (Al)-metal batteries,^50,51^ Schoetz et al.^50^ investigated chloroaluminate ionic liquid mixtures of aluminum chloride (AlCl_3_), 1-ethyl-3-methylimidazolium (EMIm) chloride, and 1-butyl-3-methylimidazolium (BMIm) chloride, as low-temperature electrolytes for Al/graphite batteries. A precipitous 61% decrease in specific capacity retention was observed between −20 °C and −40 °C, compared to a modest 8% decrease between −10 °C and −20 °C using their optimum electrolyte, AlCl_3_-EMImCl:BMImCl (2:1). A similar trend can be observed for potassium (K)-ion batteries using graphite electrodes for K^+^ cation intercalation. For example, Cheng et al.^52^ measured a significant ∼40% decrease in specific capacity between −20 °C and −30 °C compared to a ∼9% decrease between 0 °C and −20 °C using 1 M potassium bis(fluorosulfonyl)imide in 2-methyltetrahydrofuran as the electrolyte.

The precipitous drop in capacity observed below −20 °C for our variable-temperature three-electrode measurements (Figure 3) does not appear to be a consequence related to electrolyte ionic conductivity or viscosity. Luo et al.,^53^ for example, formulated electrolyte mixtures of 1 M LiPF_6_ dissolved in EC, EMC, and isobutyronitrile (iBN) as co-solvent. The addition of iBN decreased viscosity and increased ionic conductivity by weakening the Li^+^ cation affinity to EMC solvent molecules. Luo et al. measured the temperature-dependence of ionic conductivity and viscosity of 1 M LiPF_6_ in EC/EMC (3:7 (v/v)) as their control electrolyte and 1 M LiPF_6_ in EC/EMC/iBN (1:3.8:7.2 (v/v)) as their best performing electrolyte. Both the ionic conductivity and viscosity of these two electrolytes displayed smooth, monotonic changes as temperature decreased from −20 °C to −40 °C. Furthermore, the addition of iBN increased the ionic conductivity from ∼0.63 mS/cm (control electrolyte) to ∼3.98 mS/cm (best electrolyte) at −40 °C. However, galvanostatic cycling data for their LiCoO_2_/graphite pouch-cells using their best electrolyte showed a ∼30% decrease in capacity between −20 °C and −40 °C, compared to a ∼4.3% decrease between −10 °C and −20 °C. Despite improvements to electrolyte transport properties at temperatures below −20 °C, significant decreases in attainable charge capacity using graphite electrodes were still observed. This drop in capacity observed for different intercalating ions (e.g., Li,^+^ AlCl_4_^-^, K^+^) using graphite electrodes that store charge by electrochemical ion intercalation suggests that the graphite material itself fundamentally limits the attainable capacity at temperatures below −20 °C.

Interestingly, the existence of a pre-formed SEI layer on the measurement of *E*_*gr*_ manifests when the temperature decreases to −30 °C and −40 °C. Following an identical formation cycling protocol to that described above, the *E*_*gr*_ profile at −20 °C is nearly identical to the one without a traditionally formed SEI (Figure 3c). As at 20 °C, this result suggests that the presence of a SEI does not affect the measurement of Li^+^ cation intercalation and Li plating down to −20 °C. However, as temperature drops to −30 °C (Figure 3d) and −40 °C (Figure 3e), the *E*_*gr*_ profiles show a significant increase in the nucleation overpotential compared to the otherwise similar *E*_*gr*_ profiles acquired with no formation cycling. The difference between the −20 °C and −30 °C *E*_*gr*_ profile with proper formation cycling is further evidence that the graphite electrode itself appears to limit the electrochemical ion-intercalation process at temperatures below −20 °C.

**Figure 4:**
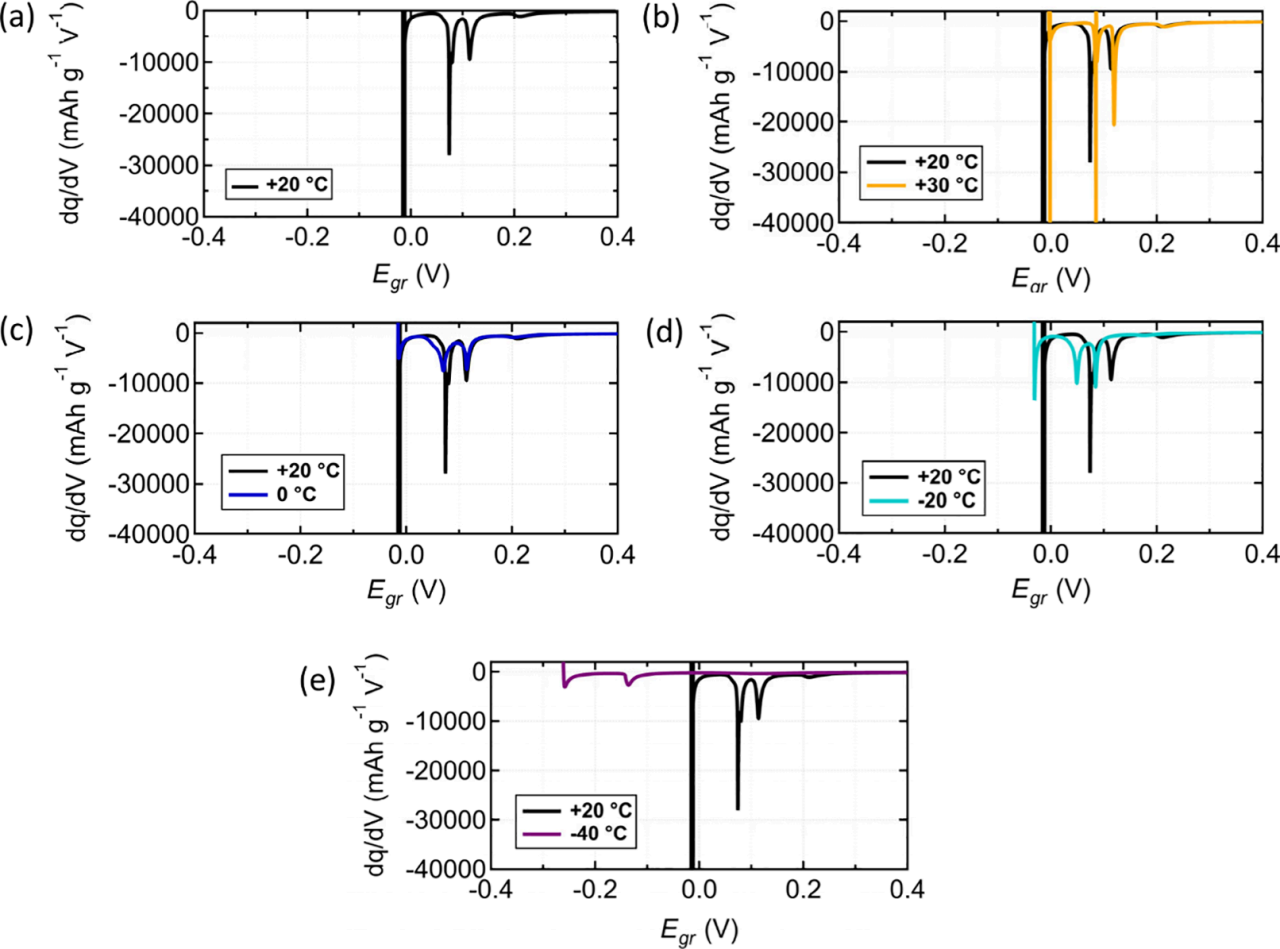
Differential capacity analysis of graphite electrode potential *E*_*gr*_ in three-electrode Li/Li/graphite cells. Variable-temperature measurements were conducted at 0.1 mA/cm^2^ at (a) 20 °C, (b) 30 °C, (c) 0 °C, (d) −20 °C, and (e) −40 °C. The data set acquired at 20 °C is included in panels b–e to highlight the effects of temperature on the Li^+^ cation intercalation and Li plating process.

Differential capacity (d*q*/d*V*) analyses were conducted on the variable-temperature *E*_*gr*_ measurements from Figure 3. The d*q*/d*V* at 20 °C (Figure 4a) shows four peaks, indicative of graphite staging and in agreement with a separately acquired two-electrode d*q*/d*V* profile conducted under identical conditions (Figure S4, Supporting Information). The d*q*/d*V* profiles were used to confirm that the Li^+^ cation intercalation reactions occurred as expected. However, as *E*_*gr*_ decreases, a vertical line in the d*q*/d*V* profile at potentials below 0 V vs. Li/Li^+^ marked the onset of Li plating.

At 0 °C (Figure 4c) and −20 °C (Figure 4d), the graphite staging peaks were shifted to lower graphite electrode potentials relative to the 20 °C d*q*/d*V* profile. At −40 °C (Figure 4e), the distinct peaks associated with Li^+^ cation intercalation and graphite staging disappear at potentials above 0 V vs Li/Li^+^, indicating that Li^+^ cation intercalation does not occur. The lack of these peaks indicates that graphite stages were not accessed during the charging process. Results from Schmitt et al.’s^54^ report on variable-temperature operando X-ray diffraction and Senyshyn et al.’s^55^ report on variable-temperature in situ neutron diffraction measurements on graphite electrodes showed that colder temperatures suppress staging.

At −40 °C, one additional peak below 0 V vs Li/Li^+^ exists, suggesting that Li^+^ cation intercalates and competes with Li plating at potentials below 0 V vs Li/Li^+^. This result is supported by Verbrugge and Koch’s^16^ paper using constant-potential holds at potentials less than 0 V vs Li/Li^+^. Furthermore, calculation of the standard reduction potential for both Li plating and Li^+^ cation intercalation as temperature varies between −40 °C and 30 °C shows that the Li plating potential is largely insensitive to changes in temperature, but the Li^+^ cation intercalation potential changes by as much as 60 mV (Text S2 and Figure S8, Supporting Information).

Our variable-temperature constant-current experiments showed clearly that at temperatures below −20 °C the accessible capacity using graphite electrodes is drastically reduced, and this indicates that a shift in the controlling mechanism for Li^+^ cation reduction is in play. In the following sections, we analyze our variable-temperature *E*_*gr*_ measurements toward this purpose.

### Mechanism of Li^+^ Intercalation and Li Plating on Graphite

**Figure 5:**
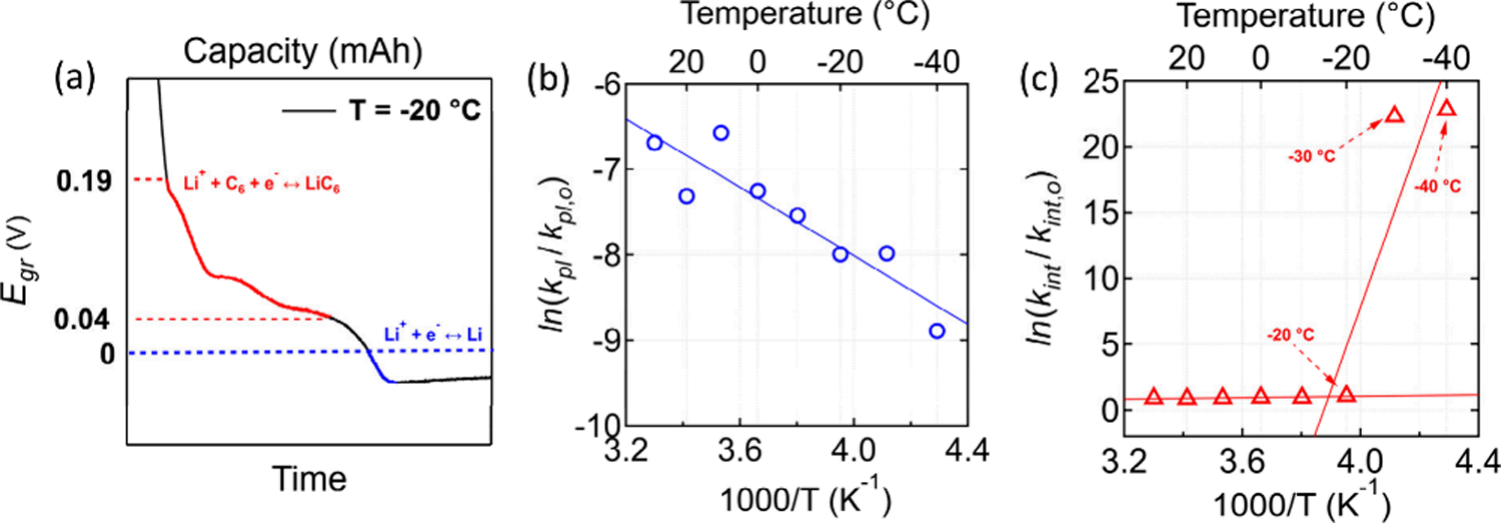
Arrhenius analysis of empirically defined rate constants for Li^+^ cation intercalation and Li plating. Temperature analysis of the graphite potential *E*_*gr*_ in three-electrode cells (Li/Li/graphite) conducted at 0.1 mA/cm^2^. (a) Definitions of Li^+^ intercalation (*t*_*int*_) and Li plating time (*t*_*pl*_) using the three-electrode *E*_*gr*_ measurement at −20 °C from Figure 3 as an example. Arrhenius plots for (b) *k*_*pl*_ and (c) *k*_*int*_.

To develop a mechanistic understanding of the Li^+^ cation reduction process on graphite, we analyzed the variable-temperature constant-current measurements quantitatively in terms of empirically defined reaction rate constants (defined below). The time duration for Li plating (*t*_*pl*_) is defined as the time from when *E*_*gr*_ = 0 V vs Li/Li^+^ reaches the local minimum, marking the onset of Li plating. Similarly, the time for electrochemical Li^+^ cation intercalation (*t*_*int*_) in graphite is defined as the time from when *E*_*gr*_ changes from 0.19 V to 0.04 V (Figure 5a). The potential range for graphite staging was determined from the baseline constant-current measurements (Figure 2). Thus, the rate constant for electrochemical Li^+^ cation intercalation is *k*_*int*_ = 1/*t*_*int*_ and for Li plating is *k*_*pl*_ = 1/*t*_*pl*_, which are defined as the inverse of time for Li^+^ intercalation and Li plating, respectively. Both *k*_*int*_ and *k*_*pl*_ represent empirical measures of the electrochemical Li^+^ cation intercalation and Li plating kinetics on graphite, respectively. The dimensions of *k*_*int*_ and *k*_*pl*_ as the inverse of time assume that Li^+^ cation intercalation and Li plating are first-order electrochemical reactions. For the purpose of this study, first-order kinetics is a reasonable starting assumption for investigating the mechanism of Li^+^ intercalation and Li plating as researchers have used Butler–Volmer and Butler–Volmer-type models, which inherently assumes that the electrochemical reaction is unimolecular.^13,44,56−58^

The temperature-dependence of *k*_*int*_ and *k*_*pl*_ can be understood using the Arrhenius equation, *k*/*k*_0_ = exp (−*E*_*A*_/*RT*), where *k*_0_ are the temperature-independent pre-exponential factors, *E*_*A*_ are the apparent activation energies for either intercalation (*E*_*A*, *int*_) or plating (*E*_*A*, *pl*_), *R* is the gas constant, and *T* is the absolute temperature. The rate constant *k*_*int*_ was fitted to extract the apparent activation energy across two temperature ranges: (i) 30 °C to −20 °C resulting in *E*_*A*, *int*_ = −2.24 ± −0.621 kJ/mol, and (ii) −20 °C to −40 °C resulting in *E*_*A*, *int*_ = −44.43 ± −12.471 kJ/mol; *negative* apparent activation energies were obtained. The fitted *E*_*A*, *int*_ for the temperature range −20 °C to −40 °C is similar in magnitude to 50 kJ/mol, as reported by Xu et al.^59^ for Li^+^ intercalation into graphite anodes. Interestingly, apparent activation energies for Li^+^ intercalation as high as 60 kJ/mol to 70 kJ/mol have also been reported.^59,60^ Xu et al. and Chekushkin et al.^61^ attributed this increased activation energy to the presence of the SEI layer. Regardless, the negative apparent activation energy suggests that electrochemical Li^+^ cation intercalation follows a multi-step process. The same temperature-dependence can also be observed even if *t*_*int*_ is expanded beginning from the start of the constant-current experiment (open circuit potential) down to the local minimum (Figure S9, Supporting Information), which would correspond physically to the charge passed for Li^+^ cation intercalation under a constant-current prior to Li plating.

The temperature dependence of *k*_*pl*_, however, shows a positive apparent activation energy *E*_*A*, *pl*_ = 16.65 ± −3.02 kJ/mol across the temperature range tested. The obtained *E*_*A*, *pl*_ for Li plating is consistent with values between 10 kJ/mol (assumed for simulations by Tewari et al.^56^) to 30 kJ/mol (experimentally determined by Aryanfar et al.^62^). From chemical kinetics, an Arrhenius process with a positive *E*_*A*_ suggests that Li plating follows a single step.

A negative apparent activation energy can occur in a two-step, pre-equilibration mechanism in a reaction network with the general form:
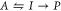
2where *A* is a reactant species, *I* is an intermediate species, and *P* is a product species. Equation 2 suggests that the kinetics of the formation of *I* can control the rate of the overall reaction (*A* → *P*).^63^ Non-electrochemical reactions can also exhibit a negative apparent activation energy, such as the heterogeneous oxidation of nitric oxide or carbon monoxide over a catalyst surface where the adsorption of intermediate species is responsible for non-Arrhenius behavior.^64−66^ Interestingly, a negative apparent activation energy for Li metal plating at low temperatures was first reported by Waldmann et al.,^67^ where Li^+^ cation adsorption was alluded to as a possible explanation for the negative apparent activation energy.

Using the general two-step, pre-equilibration mechanism (eq 2) as a model for electrochemical Li^+^ cation intercalation into graphite and Li plating using adsorbed Li^+^ cations as the intermediate species, the following reaction sequence of elementary steps is proposed:
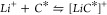
3
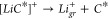
4
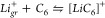
5
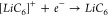
6
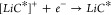
7
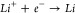
8where *Li*^+^ is a Li^+^ cation in the electrolyte, C* is a surface site on graphite, [LiC*]^+^ is a Li^+^ cation adsorbed on a surface site, *Li*_*gr*_^+^ is an intercalated Li^+^ cation between the graphene layers that has not yet adsorbed, *C*_6_ is a molecular site for adsorption and electrochemical Li^+^ cation intercalation, [*LiC*_6_]^+^ is a Li^+^ cation adsorbed on *C*_6_ that has not yet been stabilized by electron transfer, *LiC*_6_ is the product after electron transfer and represents Li^+^ electrochemically intercalated into graphite, LiC* is a neutral Li atom plated on a graphite surface site after electron transfer, and *Li* represents Li plating on Li metal during growth. Note that [*LiC*_6_]^+^ refers to a Li^+^ cation that is adsorbed on *C*_6_, i.e., a graphene interlayer, which is distinct from a Li^+^ cation adsorbed on the graphite particle surface, [LiC*]^+^, a process that also must occur prior to electrochemical Li^+^ cation intercalation.

Li^+^ cations from the electrolyte first adsorb on graphite to form [LiC*]^+^ (eq 3) prior to either intercalation (eqs 4–6) or plating (eqs 7 and 8). To the best of our knowledge, Zhu et al.^68^ first reported experimental evidence of Li^+^ cation adsorption on highly oriented pyrolytic graphite electrodes using electrochemical scanning tunneling microscopy. The surface sites C* can either be edge sites, defects, or sites along the basal plane of graphite. Edges and defects are generally understood as sites of high activity for electrochemical redox on graphite electrodes and are subjects of intense computational studies.^69−73^

**Scheme 1:**
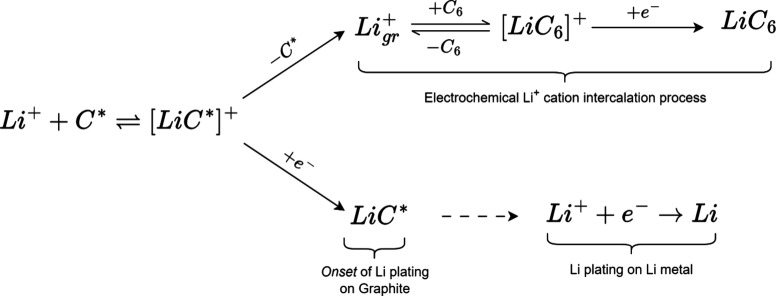
Mechanism of Electrochemical Li^+^ Cation Intercalation and Plating on Graphite

Following adsorption, an electron transfer needs to occur to stabilize the Li^+^ cation with *C*_6_ to form *LiC*_6_ (eq 6). Thus, Li^+^ intercalates first as ions (eq 4), where they then diffuse via a hopping mechanism (eq 5) until they become stabilized by the electron cloud density of *C*_6_ (eq 6).^74,75^ Therefore, *C*_6_ can be regarded as an active site for the electrochemical ion-intercalation process where Li^+^ cations adsorb/desorb (eq 5) as part of the electrochemical Li^+^ cation intercalation process during charge. Instead of intercalating, the adsorbed Li^+^ cation on C* can also reduce to form LiC*, which marks the onset of Li plating on graphite (eq 7) and occurs when the graphite potential *E*_*gr*_ is below 0 V vs Li/Li^+^. Beyond the minimum in *E*_*gr*_, the nucleated Li metal will grow (eq 8). Scheme 1 summarizes the relationships among eqs 3–8:

Scheme 1 shows that the kinetics of Li^+^ intercalation or Li plating can depend on the kinetics of Li^+^ cation adsorption on the graphite surface (eq 3), and on the kinetics of Li^+^ cation adsorption on *C*_6_ (eq 5). Electrochemical Li^+^ cation intercalation (eqs 4 and 6), and Li plating (eqs 7 and 8) are written as irreversible reactions to represent the constant-current conditions during battery charging. The negative apparent activation energies *E*_*A*_ obtained from fitting the Li^+^ intercalation rate constant *k*_*int*_ (Figure 5c) suggests that the electrochemical Li^+^ cation intercalation process is an interplay between Li^+^ cation adsorption/desorption on *C*_6_ (eq 5) followed by electron transfer (eq 6). On the other hand, the positive *E*_*A*_ for the Li plating rate constant *k*_*pl*_ (Figure 5b) suggests that Li plating follows a simple, elementary electrochemical step that requires only an electron to reduce Li^+^ cations to Li metal (eq 7). Solvent co-intercalation can be disregarded due to the formation of a primitive, SEI-like layer in these measurements (Figure S5, Supporting Information), which is known to prevent solvent co-intercalation.^76^

### Quantitative Arguments of Li^+^ Interfacial Concentration using Langmuir Adsorption Model

Both [LiC*]^+^ and [*LiC*_6_]^+^ reflect the interfacial concentration of Li^+^ cations adsorbed on graphite surface and molecular active sites for electrochemical ion-intercalation, respectively. Le Chatelier’s principle guides the equilibrium for Li^+^ cation adsorption/desorption. Because adsorption is inherently exothermic, its equilibrium with desorption shifts depending on temperature: as temperature decreases, the equilibrium for the adsorption/desorption reaction (eqs 3 and 5) shifts to the right, promoting adsorption of Li^+^ cations. Conversely, as temperature increases, the equilibrium shifts to the left facilitating the desorption process. To further understand the effect that temperature has on the interplay between Li^+^ cation adsorption/desorption, electrochemical Li^+^ cation intercalation, and Li plating, we use the Langmuir adsorption kinetic model as a physically intuitive framework to demonstrate how (i) the accessible capacity during charge depends on the ability of the Li^+^ cations to find available *C*_6_ sites for ion-intercalation, and (ii) how the overall rate of the surface reaction (either electrochemical ion-intercalation or Li plating) is subject to rate-limitations that dictate the ability of Li^+^ cations to search for and reduce on active sites. Although originally developed to model gas-phase heterogeneous catalysis and monolayer formation,^77^ the Langmuir adsorption kinetic model can be used to explain the mechanistic pathways for Li^+^ reduction on graphite, and graphite’s inability to build capacity at colder temperatures and fast charging rates.

We applied the Langmuir kinetic model to a reaction sequence comprised of Li^+^ cation adsorption/desorption on active sites (eqs 3 and 5) followed by irreversible electron transfer (eqs 6 and 7). This reaction sequence is captured by the two-step, pre-equilibration mechanism *A* ⇋ *I* → *P* (eq 1). For the electrochemical Li^+^ cation intercalation process in graphite, the rates of the three elementary steps can be written as follows:
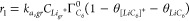
9
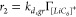
10
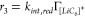
11where *r*_1_ is the rate of Li^+^ cation adsorption on *C*_6_ (eq 5), *r*_2_ is the rate of Li^+^ cation desorption from *C*_6_ (eq 5), *r*_3_ is the rate of the electron transfer step to form *LiC*_6_ (eq 6), *k*_*a*, *gr*_ is the rate constant for adsorption on C_6_, *k*_*d*, *gr*_ is the rate constant for desorption from C_6_, *k*_*int*, *red*_ is the rate constant for electron transfer, *C*_*Li*_*gr*_^+^_ is the concentration of Li^+^ cations in graphite, Γ_*C*_6__^0^ is the interfacial concentration of *C*_6_ sites for ion-intercalation or adsorption, Γ_[*LiC*_6_]^+^_ is the interfacial concentration of adsorbed Li^+^ cations on *C*_6_, *θ*_[*LiC*_6_]^+^_ is the fractional coverage of [*LiC*_6_]^+^, and *θ*_*LiC*_6__ is the fractional coverage of electrochemically intercalated Li^+^ cations. The rate constant *k*_*int*, *red*_ quantifies the kinetics of the electron transfer to [*LiC*_6_]^+^ (eq 6) and is different from the empirically defined rate constant *k*_*int*_ in the preceding section. We treat *k*_*int*_ as an overall rate constant for the electrochemical Li^+^ cation intercalation process. The mole balance on Γ_[*LiC*_6_]^+^_ can be written as
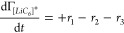
12The electron transfer to [*LiC*_6_]^+^ to form *LiC*_6_ can be treated as an irreversible reaction under galvanostatic conditions that occupies an active site *C*_6_. Therefore, a site balance must be written to describe the occupation of *C*_6_ sites either by Li^+^ cation adsorption/desorption (eq 5) or electrochemical ion-intercalation (eq 6):
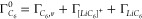
13
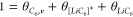
14where Γ_*C*_6_, *v*_ is the number of vacant *C*_6_ sites, Γ_*LiC*_6__ is the interfacial concentration of electrochemically intercalated Li^+^ cations, and *θ*_*C*_6_, *v*_ is the fractional coverage of the vacant *C*_6_ sites. The total number of *C*_6_ sites Γ_*C*_6__^0^ for electrochemical ion-intercalation is a material parameter that is fixed by graphite. Using the steady-state approximation (dΓ_[*LiC*_6_]^+^_/d*t* =0), the fractional coverage for [*LiC*_6_]^+^ can be expressed in terms of the fractional coverage of *LiC*_6_, the rate constants for Li^+^ cation adsorption/desorption on *C*_6_, and rate constant for electron transfer to electrochemically intercalate Li^+^ cations:
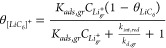
15where an equilibrium constant *K*_*ads*, *gr*_ = *k*_*a*, *gr*_/*k*_*d*, *gr*_ can be defined that characterizes the adsorption/desorption equilibrium for Li^+^ cations on *C*_6_ sites.

The fractional coverage of *LiC*_6_ (*θ*_*LiC*_6__) is a measure of the amount of capacity stored in graphite through electrochemical ion-intercalation (372 mAh/g theoretical capacity) assuming one Li^+^ cation sits on the center of a *C*_6_ ring. The decrease in Li^+^ cation storage capacity in graphite at lower temperatures, as observed in our variable-temperature three-electrode measurements (Figure 3) and d*q*/d*V* analyses (Figure 4), can be understood by examining how the temperature-dependence of *k*_*a*, *gr*_, *k*_*d*, *gr*_, and *k*_*int*, *red*_ decreases *θ*_*LiC*_6__. Assuming that *k*_*a*, *gr*_ is independent of temperature,^78^ and *k*_*d*, *gr*_, *k*_*int*, *red*_ follows Arrhenius behavior and decreases as temperature decreases, the expression for *θ*_[*LiC*_6_]^+^_ (eq 15) demonstrates that the accessible capacity in graphite *θ*_*LiC*_6__ decreases at colder temperatures. Furthermore, there is an interesting inverse relationship between *θ*_[*LiC*_6_]^+^_ and *θ*_*LiC*_6__; a decrease in *θ*_*LiC*_6__ increases *θ*_[*LiC*_6_]^+^_. This result suggests an inability to electrochemically reduce [*LiC*_6_]^+^ to *LiC*_6_ at colder temperatures.

To better understand this interplay between [*LiC*_6_]^+^, *LiC*_6_, and vacant *C*_6_ sites under galvanostatic charging conditions, the overall reaction rate for electrochemical Li^+^ cation intercalation (*r*_*int*_) can be written as
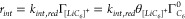
16The fractional coverage *θ*_[*LiC*_6_]^+^_ (eq 15) can be inserted into eq 16 to yield
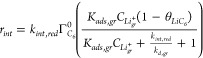
17The overall rate *r*_*int*_ can be simplified further by defining *k*_*int*_ as a collection of terms that relates the kinetic rate constants for Li^+^ cation adsorption/desorption on *C*_6_ and electron transfer at *C*_6_ sites:
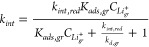
18By substituting the expression (1 – *θ*_*LiC*_6__) = *θ*_*C*_6_, *v*_ + *θ*_[*LiC*_6_]^+^_ (eq 14) into eq 17 and collecting the rate constants (eq 18), the overall electrochemical Li^+^ cation intercalation rate becomes
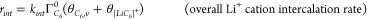
19

Two rate limitations emerge depending on the relative magnitudes of the elementary rate constants: (i) adsorption-limited (*k*_*int*, *red*_ ≫ *k*_*a*, *gr*_) and (ii) surface reaction-limited (*k*_*int*, *red*_ ≪ *k*_*d*, *gr*_). The rate constant *k*_*int*_ (eq 18) and thus the overall rate for electrochemical Li^+^ cation intercalation (eq 19) can be simplified depending upon the rate-limiting regime:
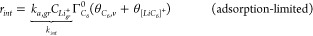
20
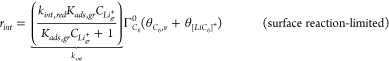
21The temperature-dependences of *k*_*a*, *gr*_, *k*_*d*, *gr*_, *k*_*int*, *red*_ and the relationship established between *θ*_*LiC*_6__ and *θ*_[*LiC*_6_]^+^_ (eq 15) described above can be used to show how the accessibility of vacant *C*_6_ sites captured by *θ*_*C*_6_, *v*_ affects the electrochemical Li^+^ cation intercalation process under a constant *r*_*int*_, which is directly established by constant-current density under galvanostatic conditions. The mechanism by which the Li^+^ cations’ ability to access vacant *C*_6_ sites for Li^+^ adsorption/desorption and electrochemical reduction during charge depends on the relative magnitude of the electron transfer kinetics (*k*_*int*, *red*_) with the rates of Li^+^ cation adsorption/desorption on *C*_6_ (*k*_*a*, *gr*_, *k*_*d*, *gr*_).

Under the adsorption-limited regime (eq 20), the electron transfer step is fast relative to the rate of Li^+^ cation adsorption on *C*_6_ such that a buildup of adsorbed Li^+^ cations [*LiC*_6_]^+^ is discouraged. The graphite potential *E*_*gr*_ at −20 °C suggests that the electrochemical Li^+^ cation intercalation process follows an adsorption-limited charging regime. This was evidenced by the plateaus for graphite staging (Figure 3a) and peaks in d*q*/d*V* profiles (Figure 4d), which shows that [*LiC*_6_]^+^ can electrochemically reduce to store charge at temperatures between 30 °C and −20 °C. The reduction to *LiC*_6_ decreases *θ*_[*LiC*_6_]^+^_, which forces *θ*_*C*_6_, *v*_ to decrease in the adsorption-limited rate expression (eq 20) to maintain a constant *r*_*int*_. The measured capacity at −20 °C was within order of magnitude to graphite’s theoretical capacity, which suggests that the decrease in *θ*_*C*_6_, *v*_ is due to the occupation of electrochemically reduced Li^+^ cations on *C*_6_ sites (LiC_6_). It is well-understood that Li^+^ cations undergo a solid-state diffusion process where the Li^+^ cations between graphite layers hop from *C*_6_ to another to electrochemically reduce and reach graphite’s theoretical capacity.^75^ Therefore, under 0.1 mA/cm^2^ and temperatures from 30 °C down to −20 °C, the accessible capacity is not limited by the ability of Li^+^ cations to desorb from *C*_6_.

For the surface reaction-limited regime (eq 21), the rate of electron transfer to [*LiC*_6_]^+^ is slow relative to the rate of Li^+^ cation adsorption on *C*_6_. At −30 °C and −40 °C, the precipitous drop in measured capacity can be attributed to a decrease in the electron transfer kinetics, resulting in a charging process where (i) a buildup of [*LiC*_6_]^+^ polarizes the graphite potential down to 0 V vs. Li/Li^+^ and below (similar to an electric double-layer capacitor), and (ii) a decreased rate of Li^+^ cation desorption from *C*_6_ (eq 5) reduces the ability of Li^+^ cations to hop from one *C*_6_ site to another to electrochemically intercalate at colder temperatures. Thus, the decrease in *θ*_*C*_6_, *v*_ under a surface reaction-limited kinetic regime is due to increases in the fractional coverage of [*LiC*_6_]^+^, and a simultaneous decrease in the rate of Li^+^ cation desorption that restricts access to vacant *C*_6_ sites during charge. The lack of significant plateaus in *E*_*gr*_ (Figure 3a), and peaks in the d*q*/d*V* plot for −40 °C (Figure 4e) is evidence for graphite’s inability to store charge through electrochemical ion-intercalation.

Thus, the transition in the graphite potential *E*_*gr*_ between −20 °C and −30 °C can be attributed to a change in the rate-limitation from adsorption-limited to surface reaction-limited for the electrochemical ion-intercalation process, as indicated by the change in slope in the Arrhenius plot for *k*_*int*_ (Figure 5c).

An analysis for the onset of Li plating on graphite surface cites C* (eqs 3 and 7) results in a similar set of equations. The fractional coverage of adsorbed Li^+^ cations on C* (*θ*_[LiC*]^+^_) can be written as
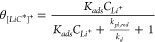
22where *K*_*ads*_ = *k*_*a*_/*k*_*d*_ is the equilibrium constant, *k*_*a*_ is the rate constant for Li^+^ cation adsorption on C*, *k*_*d*_ is the rate constant for Li^+^ cation desorption from C*, and *k*_*pl*, *red*_ is the rate constant for electron transfer to plate Li metal (eq 7). A site balance on the total number of C* sites (Γ_C*_^0^) yields
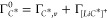
23
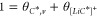
24where Γ_C*, *v*_ is the interfacial concentration of vacant C* sites, Γ_[LiC*]^+^_ is the interfacial concentration of adsorbed Li^+^ cations on C*, *θ*_C*, *v*_ is the fractional coverage of vacant C* sites, and *θ*_[LiC*]^+^_ is the fractional coverage of adsorbed Li^+^ cations on C*. Unlike the site balance for electrochemical Li^+^ cation intercalation (eq 13), the site balance for the onset of Li plating does not include the interfacial concentration of LiC*. The formation of LiC* generates additional surface sites for Li metal growth (eq 8) under galvanostatic conditions. Thus, the total number of sites C* is conserved for analyzing the onset of Li plating on graphite and is a parameter fixed by graphite. Using the same temperature-dependences for *k*_*a*_, *k*_*d*_, and *k*_*pl*, *red*_ from the analysis of electrochemical Li^+^ cation intercalation above, the expression for *θ*_[LiC*]^+^_ (eq 22) shows that the fractional coverage of adsorbed Li^+^ cations on C* sites increases as temperature decreases. Physically, the lower temperature pushes the equilibrium of the adsorption/desorption reaction (eq 3) to the right.

To better understand how increases in *θ*_[LiC*]^+^_ affect the rate of Li plating onset (*r*_*pl*_), a similar equation to the overall rate of electrochemical Li^+^ cation intercalation (eq 17) can be derived:
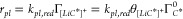
25
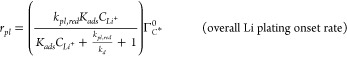
26The constants *k*_*a*_, *K*_*ads*_, and *k*_*pl*, *red*_ can be collected into an overall rate constant *k*_*pl*_:
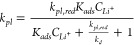
27The overall rate of Li plating onset (eq 26) can be simplified into an adsorption-limited regime (*k*_*pl*, *red*_ ≫ *k*_*a*_) and surface reaction-limited regime (*k*_*pl*, *red*_ ≪ *k*_*d*_):
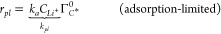
28
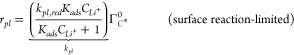
29

The Arrhenius analysis of *k*_*pl*_ (Figure 5b) suggests that the onset of Li plating follows a surface reaction-limited regime (eq 29) due to the rate expression’s dependence on *k*_*pl*, *red*_. As temperature decreases, the overall rate constant *k*_*pl*_ in the surface reaction-limited expression decreases as temperature decreases. This forces *r*_*pl*_ to remain constant by decreasing the number of vacant C* sites for Li^+^ cation adsorption and Li plating, and increasing Γ_[LiC*]^+^_ due to the decreased temperature (eqs 22 and 23). This result suggests that the total number of graphite surface sites C* is occupied by [LiC*]^+^ during the Li nucleation process, which results in significant electrode polarization and a near vertical decrease in graphite potential *E*_*gr*_ towards and below 0 V vs. Li/Li^+^ from 30 °C down to −40 °C (Figure 3a,b). In our variable-temperature graphite potential measurements, the nucleation overpotential for Li metal increased as temperature decreased, which suggests that Li metal nucleates as densely packed Li particles on the graphite surface. The nucleation overpotential for Li plating increases significantly at −30 °C and −40 °C (Figure 3a, d-e), indicating that an energy penalty was incurred for nucleating densely packed Li particles on the graphite surface. Therefore, a higher occupation of C* sites by adsorbed Li^+^ cations is required to meet the energy requirements to nucleate and plate Li metal on graphite at lower temperatures.

### Variable Current–Density Measurements of Li Plating on Graphite

**Figure 6:**
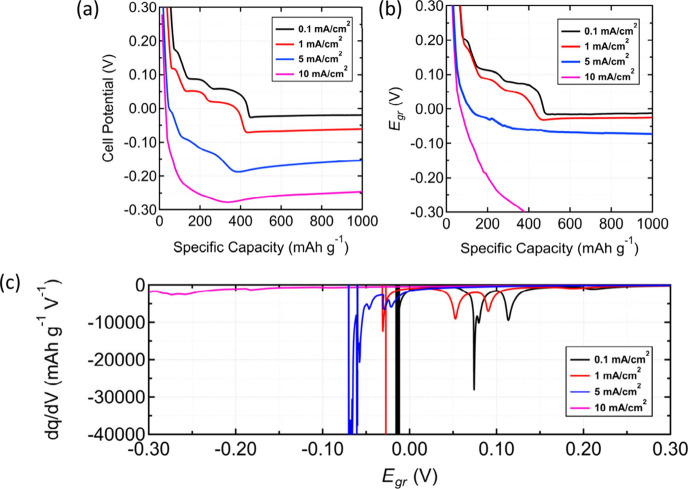
Effect of changing current density from 0.1 to 10 mA/cm^2^ at 20 °C without formation cycling. (a) Two-electrode Li/graphite cell potential profiles. (b) Three-electrode Li/Li/graphite measurements of graphite potential *E*_*gr*_. (c) d*q*/d*V* analyses of the three-electrode *E*_*gr*_ data. The d*q*/d*V* profile at 0.1 mA/cm^2^ is identical to the one in Figure 4a. The corresponding C-rates for 0.1 mA/cm^2^, 1 mA/cm^2^, 5 mA/cm^2^, and 10 mA/cm^2^ are ∼C/24, ∼C/2.4, ∼2.06C, and ∼4.13C, respectively.

To investigate the effect of increasing current, both two- and three-electrode cells were charged at constant current densities ranging from 0.1 mA/cm^2^ (∼C/24) to 10 mA/cm^2^ (∼4.13C) at a constant temperature of 20 °C (Figure 6). Qualitatively, we observed that as current increases from 0.1 mA/cm^2^ to 10 mA/cm^2^, both the two-electrode cell potential (Figure 6a) and three-electrode graphite *E*_*gr*_ profiles (Figure 6b) exhibited a near immediate decrease in potential similar to the −20 °C to −30 °C and −40 °C measurements (Figure 3a) above. The d*q*/d*V* analysis (Figure 6c) also shows the expected shift in peaks associated with graphite staging. Interestingly, the d*q*/d*V* for 5 mA/cm^2^ shows several smaller peaks below 0 V indicating that even at a moderately faster charging current, electrochemical Li^+^ cation intercalation can still occur albeit at electrode potentials <0 V within the regime for Li plating. At 10 mA/cm^2^, the d*q*/d*V* profile shows a flat line, indicating that no Li^+^ cation intercalation has occurred at this charging current.

From the discussion above, the act of increasing the charging current (i.e., increasing *r*_*int*_) switches the kinetic regime from adsorption-limited to surface reaction-limited. At 0.1 and 1 mA/cm^2^, electrochemical Li^+^ cation intercalation is still fast enough to reduce [*LiC*_6_]^+^ to *LiC*_6_ evidenced by the distinct plateaus in potential and peaks in their d*q*/d*V* profiles. However, at 10 mA/cm^2^, the electrochemical Li^+^ cation intercalation process can no longer deplete [*LiC*_6_]^+^ at a sufficient rate, resulting in a buildup of Li^+^ cations adsorbed on *C*_6_ sites, which polarizes the electrode potential to 0 V vs Li/Li^+^ and within the regime to plate Li metal.

Scheme 1 and the rate-limitations (eqs 20, 21, 28, and 29) derived based on constant-current measurements of the graphite potential implicitly assumed that the Li^+^ cation reduction process on graphite is kinetically-limited and not mass transport-limited. The limiting current (*i*_*l*_) assuming mass transport-limitations through the electrolyte was calculated as *i*_*l*_ ≈ 16.08 mA/cm^2^ (Text S3, Supporting Information). Thus, for the current densities tested above, it is reasonable to assume that transport through the electrolyte does not limit the Li^+^ cation reduction process on graphite. Furthermore, mass-transfer limitations typically manifest at higher temperatures for (electro)chemically-reacting systems due to the lower activation energy for diffusion relative to the activation energy for chemical reaction. Thus, (electro)chemical reaction kinetics are more sensitive to temperature changes than mass transport rates.^79,80^

## Conclusion

To enable Li-ion batteries to operate at low temperatures and high charging currents, a mechanistic understanding of how Li plates on graphite is essential for developing mitigation strategies. In this work, we present a kinetic mechanism that includes Li^+^ cation adsorption on *C*_6_ active sites on the graphene interlayers as a critical intermediate step prior to either the electrochemical intercalation of Li^+^ cations (i.e., after electron transfer) or Li plating. Both two- and three-electrode cells using Li metal as the counter/reference electrode and graphite as the working electrode were used to galvanostatically force Li metal to plate on graphite at varying temperatures (30 °C and −40 °C) and charging currents (0.1 mA/cm^2^ to 10 mA/cm^2^). Three-electrode measurements of the graphite potential reveal a sharp decrease in the extent of electrochemical Li^+^ cation intercalation and thus attainable capacity below −20 °C, as observed for other ions intercalating into graphite, suggesting that the graphite limits the quantity of charge stored below this temperature. The temperature-dependence on empirically defined rate constants revealed typical Arrhenius behavior for Li plating and non-Arrhenius behavior for electrochemical Li^+^ cation intercalation, pointing to a Li^+^ reduction mechanism where electrochemical Li^+^ cation intercalation is a multi-step process and Li plating is a unimolecular single-step process. Variable-rate measurements on two- and three-electrode cells show that the potential profiles for the faster rates exhibit key similarities with those at lower temperatures. Ultimately, the mechanistic insights provide a kinetic framework for understanding electrochemical Li^+^ cation intercalation into, and Li plating on, graphite electrodes during charge. These insights can be leveraged for designing Li-ion battery anodes, electrolytes, and their respective interfaces and interphases, as well as controlling electrochemical cycling protocols and battery temperature regimes, to mitigate Li plating reactions.

## Supporting Information Available

Fomation cycling cell potential profile, differential capacity analyses calculation details, differential capacity analysis, cell potential profiles, comparison of cell potential measurements, additional Arrhenius plot for *k*_*int*_, calculation details for standard potentials, calculation details for limiting current (PDF)
