# Supplementary material for: Mechanistic Understanding of Lithium-Ion Adsorption, Intercalation, and Plating during Charging of Graphite Electrodes
Source: ACS Electrochem. 2025 Apr 22;1(5):574–87. doi: 10.1021/acselectrochem.4c00079 (PMC12051203; doi:10.1021/acselectrochem.4c00079)
Supplement: Supplementary file 1 — ec4c00079_si_001.pdf [file ec4c00079_si_001.pdf]

Supporting Information

Mechanistic Understanding of Lithium-Ion Adsorption, Intercalation, and Plating during Charging of Graphite Electrodes

Brian Chen[1], Niya Hope-Glenn[1,†], Amanda Wright[1,†], Robert J. Messinger[1,*], Alexander Couzis[1,*]

[1]*Department of Chemical Engineering, The City College of New York, CUNY, 160 Convent Avenue, New York, New York, 10031, United States*

[†]N.HG. and A.W. contributed equally

[*]Corresponding Authors: rmessinger@ccny.cuny.edu, acouzis@ccny.cuny.edu

Table of Contents

Figure S1. Typical formation cycling protocol for two-electrode NMC811/graphite cell

Text S1. Calculation of differential capacity (dq/dV) and smoothing

Figure S2. Effect of data points used for moving average and resolution of dq/dV plots

Figure S3. Effect of applying Savitzky-Golay smoothing on resolution of dq/dV plots

Figure S4. Typical dq/dV plot for two-electrode Li/graphite cell at 0.1 mA/cm^2

Figure S5. Two- and three-electrode cell potential profiles showing onset of SEI formation

Figure S6. Comparison of two- and three-electrode cell potential measurements between 30 °C and -40 °C

Figure S7. Three-electrode cell potential and Li metal counter electrode potentials measured between 30 °C and -40 °C

Text S2. Calculation of standard potentials for Li plating and Li^+ intercalation

Figure S8. Standard potential for Li plating and Li^+ intercalation between 30 °C and -40 °C

Figure S9. Arrhenius plot for alternative definition of k_{int}

Text S3. Calculation of limiting current through electrolyte

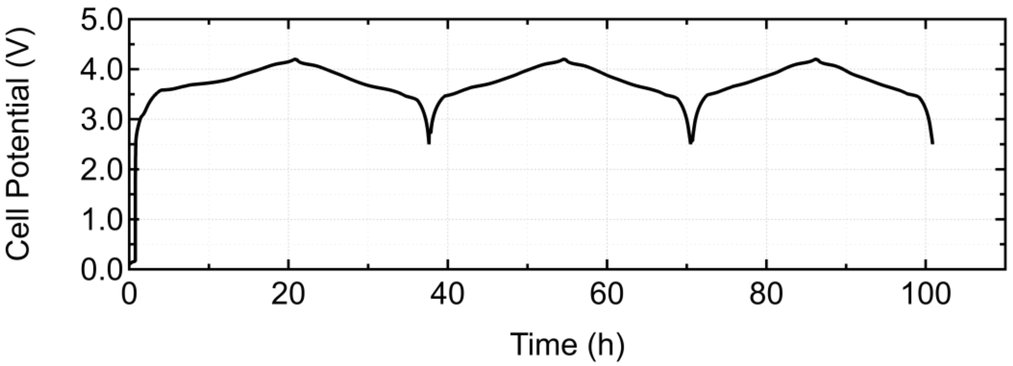

Figure S1. Formation cycling profile for a two-electrode NMC811/graphite cell at room temperature to build up a solid electrolyte interphase layer on graphite prior to harvesting and placement in a three-electrode Li/Li/graphite cell.

__Supporting Text and Calculations__

Text S1. Calculation of Differential Capacity (dq/dV) and Smoothing

Differential capacity (dq/dV) was calculated using the moving average (Eq. S1):

$$\frac{dq}{dV} = \frac{\sum_{n}\big((V - \bar{V})(q - \bar{q})\big)}{\sum_{n}(V - \bar{V})^2} \qquad (S1)$$

where n is the number of data points (both voltage and specific capacity) considered for the average, q is the specific capacity, V is voltage measurements, $\bar{V}$ is the average voltage taken over n data points, and $\bar{q}$ is the average specific capacity taken over n data points. The resolution of dq/dV depends on n, which can affect the peaks calculated by the moving average (Figure S2).

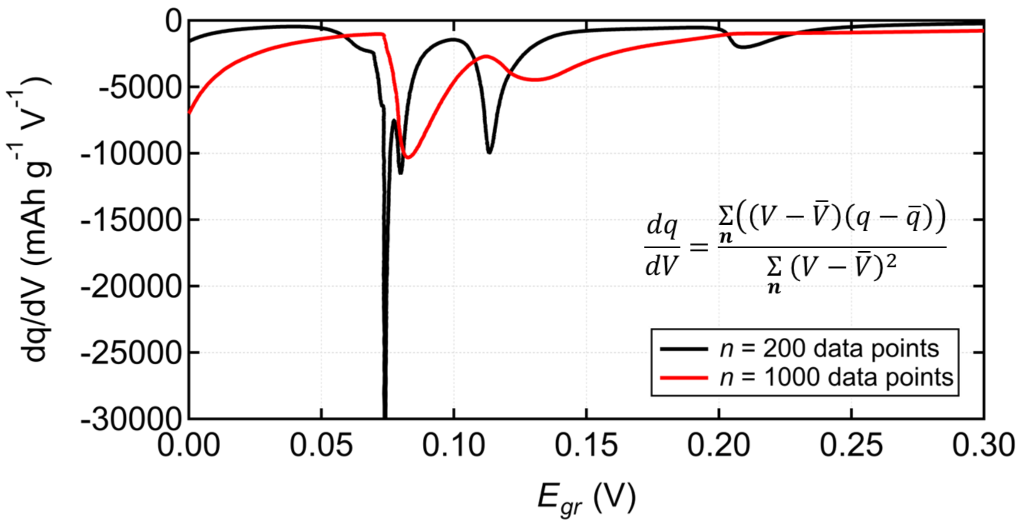

Figure S2. Resolution of the differential capacity (dq/dV) plot depends on the number of data points (n) used. Using the three-electrode graphite electrode potential (E_{gr}) measured at 20 °C and 0.1 mA/cm^2 (Figure 2a, Main Text) as an example, dq/dV is calculated and plotted for 200 data points (solid black), and 1000 data points (solid red) using the moving average formula (Eq. S1).

The resolution of the dq/dV depends on the resolution of the raw constant-current measurement. Particularly at lower temperatures, the constant-current measurements of electrode potential were noticeably noisier (Figure S3a,b and Figure 3, Main Text). Prior to calculating dq/dV, a Savitzy-Golay smoothing function on MATLAB was applied on raw voltage data first followed by dq/dV on the smoothed voltage data (Figure S3c). The smoothing procedure shows that the dq/dV calculation on the smoothed voltage data was able to capture the essential features for the Li^+ cation reduction process on charge for Li/graphite cell.

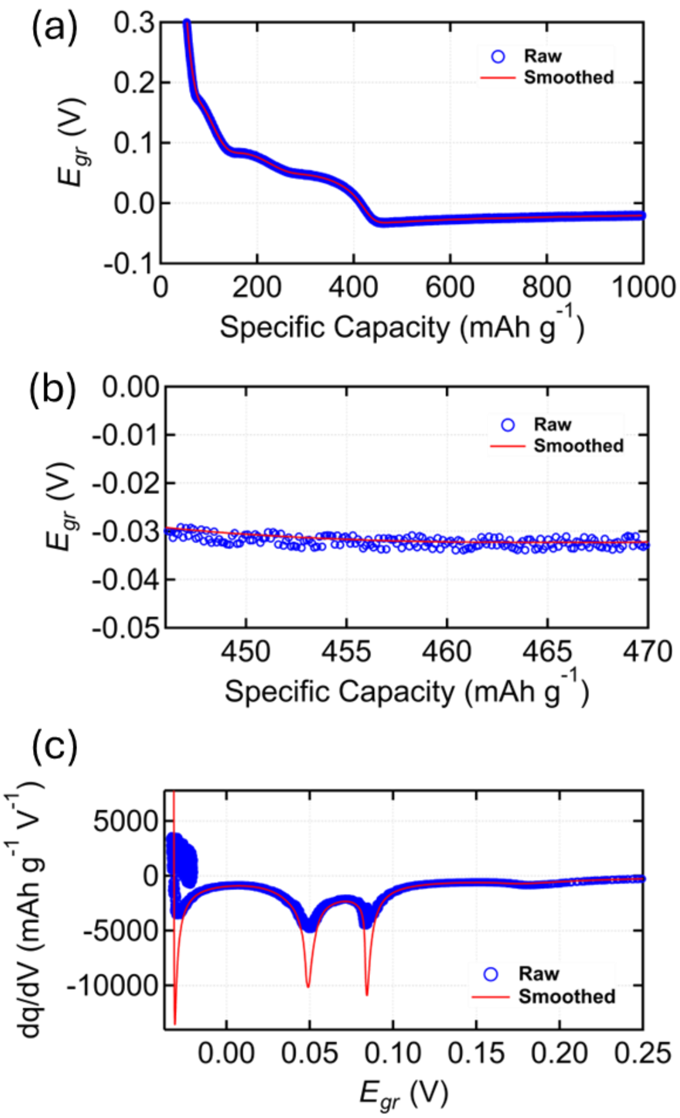

Figure S3. Using the three-electrode Li/Li/graphite electrode potential (E_{gr}) measured at -20 °C and 0.1 mA/cm^2 (Figure 3a, Main Text) as example data**, (a)** a Savitzky-Golay smoothing function using a 200 data point span and n = 1 polynomial applied to the raw (blue circles) data compared to the smoothed result (red line), **(b)** zoomed-in version of panel (a) for better visualization of the improvement that the Savitzky-Golay smoothing provides. **(c)** comparison of the dq/dV calculated on the *raw* E_{gr} data in comparison to the dq/dV calculated on the *smoothed* E_{gr}.

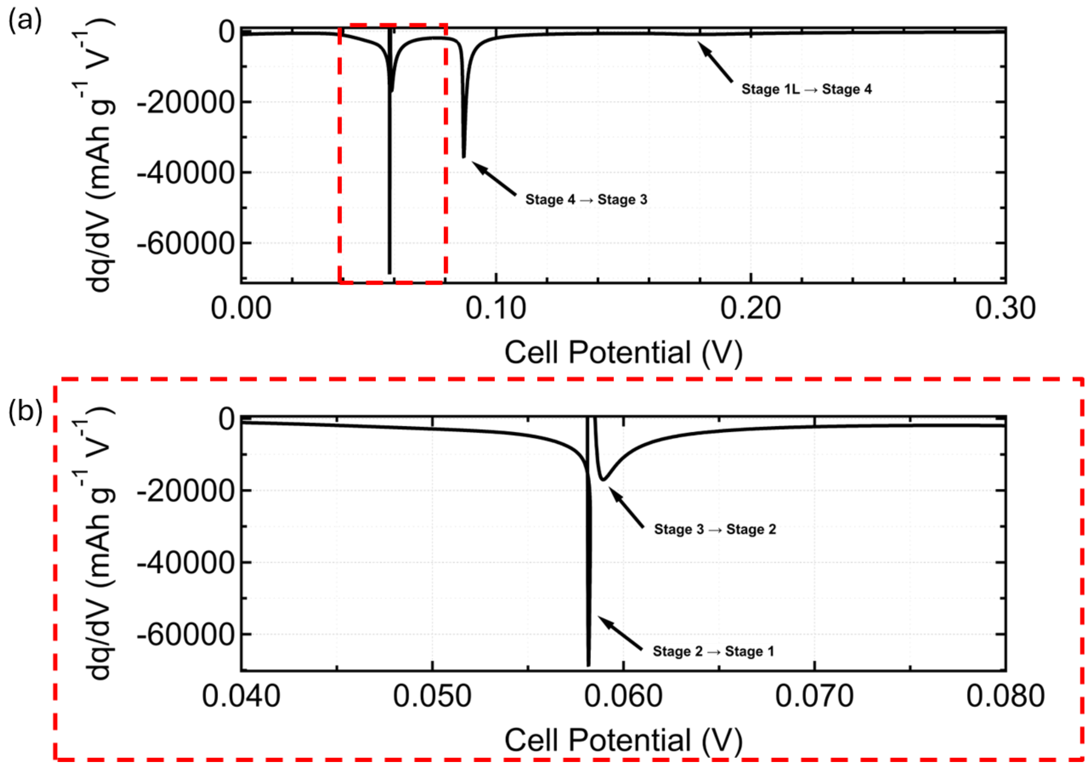

Figure S4. dq/dV plot calculated from the two-electrode Li/graphite cell potential measured at 20 °C and 0.1 mA/cm^2 (Figure 2a, Main Text). **(a)** The dq/dV was calculated using n = 200 data points. The stage 1L → stage 4, stage 4 → stage 3, stage 3 → stage 2, and stage 2 → stage 1 transitions are shown corresponding to peaks shown between 0 and 0.3 V. **(b)** The inset shows the zoomed in region between 0.05 V and 0.07 V showing the stage 3 → stage 2 and stage 2 → stage 1 transitions. The phase transitions of graphite as Li^+ intercalates were elucidated by Dahn et al.[1,2] dq/dV was obtained on two-electrode cell potential data smoothed with a Savitzy-Golay function (200 data points, polynomial order 1).

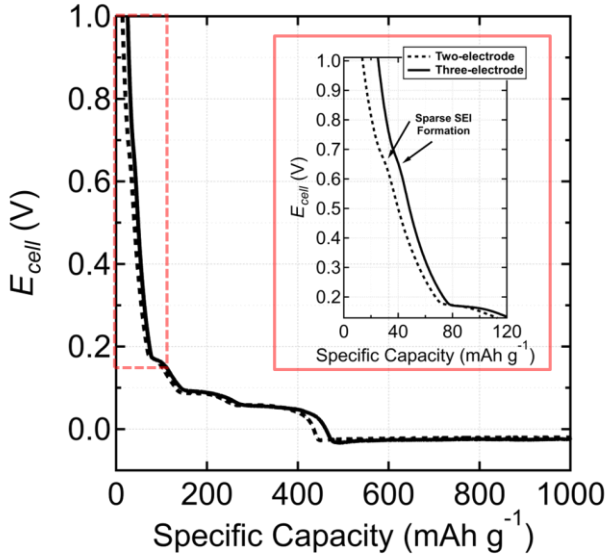

Figure S5. Expanded view of cell potential data of two- and three-electrode Li/graphite cells (Figure 2a, Main Text) with an inset showing the onset of solid electrolyte interphase formation on graphite at ~0.7 V as supported by Dahn et al.[1,2]

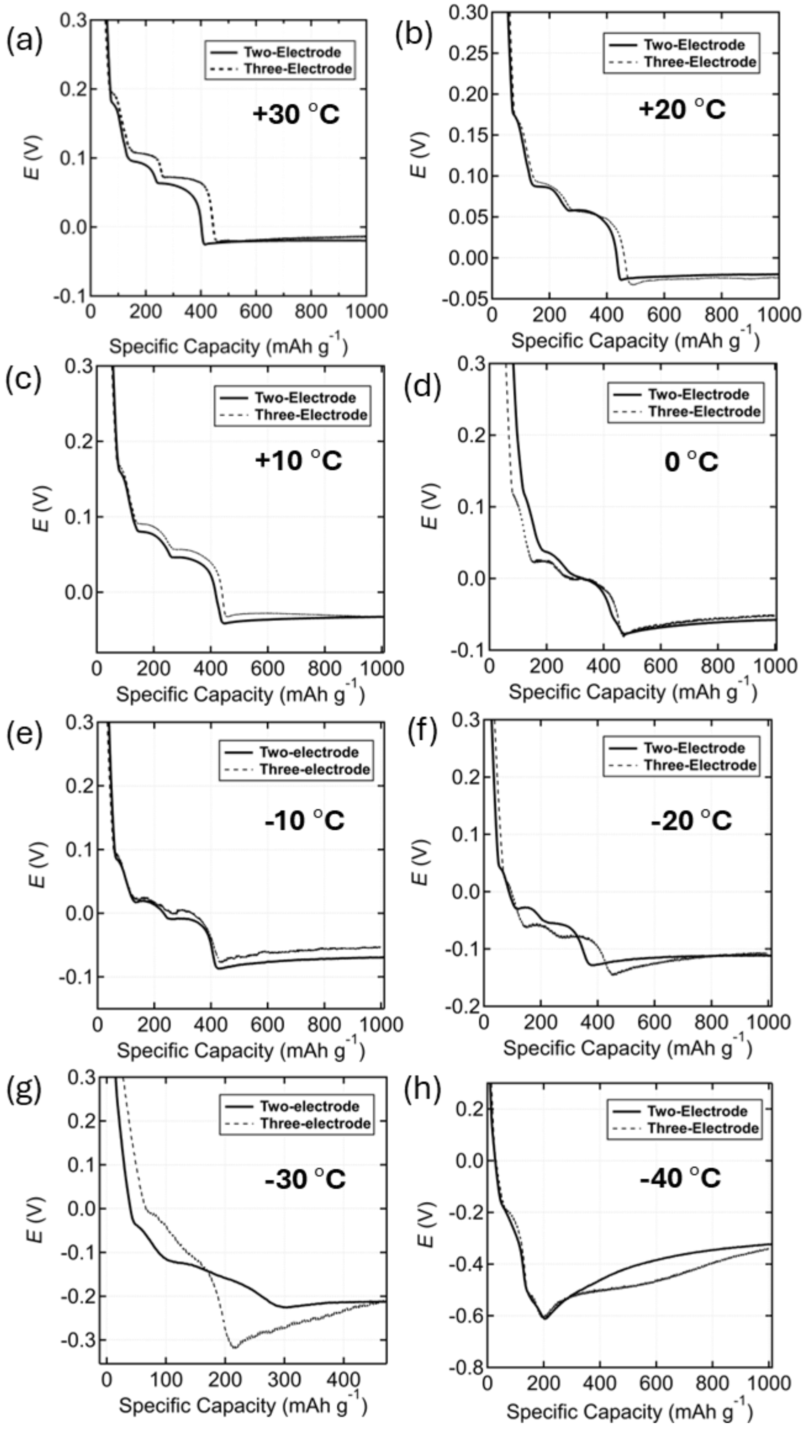

Figure S6. Two-electrode Li/graphite cell potentials in comparison to three-electrode $E_{cell} = E_{gr} - E_{Li}$ measurements. **(a)-(h)** show the comparison of the potential responses between 30 °C and -40 °C at a constant-current density of 0.1 mA/cm^2.

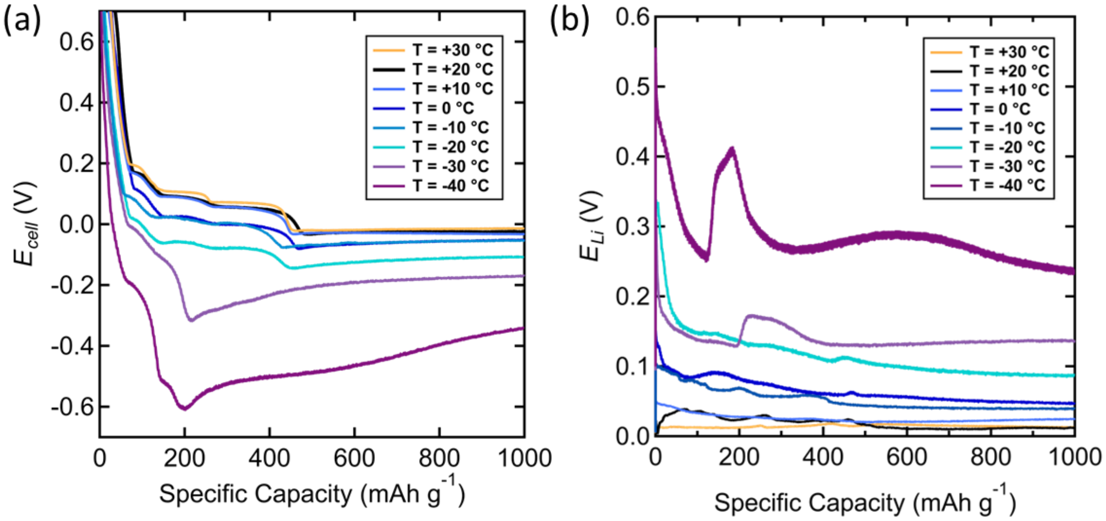

Figure S7. Galvanostatic three-electrode Li/Li/graphite measurements from 30 °C down to -40 °C showing **(a)** the full-cell Li/graphite electrode potential difference (E_{cell}), and **(b)** Li metal counter electrode vs. Li metal reference (E_{Li}). We note that as temperature decreases, the overpotential for E_{Li} increases, contributing to the electrode potential cell response E_{cell}.

Supporting Text & Calculations

Text S2. Calculation of Standard Potentials for Li plating and Li intercalation

Temperature changes can shift the standard potential (U^{θ}). Following the method from Fuller and Harb,[3] we demonstrate that the standard potentials for Li plating on graphite (U^{θ}_{Li}) and ($U^{\theta}_{LiC_6}$) can shift within the temperature range -40 °C and +30 °C as measured in our three-electrode measurements (Figure 3, Figure 4, Main Text).

Defining the full-cell reactions

In a Li/graphite electrode couple (i.e., a two-electrode cell), the standard potentials (defined at T = 298 K, P = 1 atm) for Li^+ intercalation into graphite (C_6) to form LiC_6 (stage 1) is:

$$U^{o}_{LiC_6} = -2.84\, V\ vs. SHE \tag{S2a}$$

and for Li electroplating:

$$U^{o}_{Li} = -3.04\, V\ vs. SHE \tag{S2b}$$

Both standard potentials are in reference to the standard hydrogen electrode (SHE). If Li metal is a counter electrode as is the case for our galvanostatic measurements, then the standard cell potential (U^{θ}_{cell}) can be defined by the difference between the half-cell reactions considered on graphite working electrode, either Li plating or Li^+ intercalation, and the half-cell reaction at the Li metal counter electrode ($Li^+ + e^- \leftrightharpoons Li$):

$$U^{\theta}_{cell,LiC_6} = \underbrace{-2.84\, V}_{U^{o}_{LiC_6}} - \underbrace{(-3.04\, V)}_{U^{o}_{Li}} = 0.20\, V \tag{S3a}$$

$$U^{\theta}_{cell,Li} = \underbrace{-3.04}_{U^{o}_{Li}} - \underbrace{(-3.04\, V)}_{U^{o}_{Li}} = 0\, V \tag{S3b}$$

Thus, $U^{\theta}_{LiC_6}$ and U^{θ}_{Li} are 0.20 V and 0 V, respectively. The reference electrode is subtracted out when considering the potential difference between half-cell reactions referenced to the same electrode.

U^{θ} can change as either temperature increases or decreases and is captured with the Gibbs-Helmholtz relation:

$$\frac{\partial}{\partial T}\left(\frac{U}{T}\right) = \frac{\Delta H(T)}{nFT^2} \tag{S4}$$

where U is the potential, T is the absolute temperature, $\Delta H(T)$ is the enthalpy of formation, n is the number of electrons transferred, and F is the Faraday's constant. The Gibbs-Helmholtz relation can be applied to both $U^{\theta}_{LiC_6}$ and U^{θ}_{Li}.

We first note that $\Delta H(T)$ is related to the constant-pressure heat capacity (C_P) by:

$$\Delta H(T) - \Delta H(T_o) = \int_{T_o}^{T} \Delta C_P \, dT \qquad (S5)$$

where T_o is a reference temperature, typically taken to be 298 K (standard temperature), and $\Delta H(T_o)$ is the standard enthalpy of formation. The heat capacity C_P is typically expressed by a Shomate polynomial of the form:

$$C_P(T) = A + BT^2 + CT^3 + DT^4 - ET^{-2} \qquad (S6)$$

where $A, B, C, D,$ and E are coefficients to the Shomate polynomial and are tabulated comprehensively for a variety of elements and molecules by the National Institute of Standards and Technology (NIST).[4] The heat capacity coefficients for Li metal and graphite are tabulated below:

Table S1. Heat capacity coefficients for lithium metal

**Phase**	$A\left(\frac{J}{mol\,K}\right)$	$B\left(\frac{J}{mol\,K^2}\right)$	$C\left(\frac{J}{mol\,K^3}\right)$	$D\left(\frac{J}{mol\,K^4}\right)$	$E\left(\frac{J\,K}{mol}\right)$
Liquid	32.469	-2.635	-6.327	4.230	0.00568
Solid	169.552	-882.711	1977.438	-1487.312	-1.609

Table S2. Heat capacity coefficients for graphite (C_6)

**Phase**	$A\left(\frac{J}{mol\,K}\right)$	$B\left(\frac{J}{mol\,K^2}\right)$	$C\left(\frac{J}{mol\,K^3}\right)$	$D\left(\frac{J}{mol\,K^4}\right)$	$E\left(\frac{J\,K}{mol}\right)$
Liquid	-	-	-	-	-
Solid	-	8.729×10^{-4}	6.27×10^{-6}	6.309×10^{-9}	-

Since A and E are not reported for graphite, the calculation for the standard potential will assume a $C_P(T)$ using B, C, and D.

The change in heat capacity (ΔC_P) represents the difference in heat capacity between products and reactants. ΔC_P can be expressed as the differences of the heat capacity coefficients between product and reactants:

$$\Delta C_P(T) = \Delta BT^2 + \Delta CT^3 + \Delta DT^4 \qquad (S7)$$

Integrating Eq. S5 with Eq. S7 as the heat capacity yields:

$$\Delta H(T) = \Delta H(T_o) + \frac{\Delta B}{2}(T^2 - T_o^2) + \frac{\Delta C}{3}(T^3 - T_o^3) + \frac{\Delta D}{4}(T^4 - T_o^4)$$

and upon plugging into the Gibbs-Helmholtz relation (Eq. S4), the following equations for temperature-dependent standard potentials for Li plating and Li^+ intercalation are:

$$U_{Li}^{\theta}(T) = \left(\frac{T}{T_o}\right) U_{Li}^{o} + \frac{T}{nF}\left[\Delta H_{f,Li}^{o}\left(\frac{1}{T_o} - \frac{1}{T}\right) + \Delta B\left(\frac{T}{2} + \frac{T_o^2}{2T} - T_o\right) + \Delta C\left(\frac{T^2}{6} + \frac{T_o^3}{3T} - \frac{T_o^2}{2}\right) + \Delta D\left(\frac{T^3}{12} + \frac{T_o^4}{4T} - \frac{T_o^3}{3}\right)\right] \quad (S8a)$$

$$U_{LiC_6}^{\theta}(T) = \left(\frac{T}{T_o}\right) U_{LiC_6}^{o} + \frac{T}{nF}\left[\Delta H_{f,LiC_6}^{o}\left(\frac{1}{T_o} - \frac{1}{T}\right) + C'_{P,LiC_6}\left(\frac{1}{T_o} - \frac{1}{T}\right) + \sigma B\left(\frac{T}{2} + \frac{T_o^2}{2T} - T_o\right) + \sigma C\left(\frac{T^2}{6} + \frac{T_o^3}{3T} - \frac{T_o^2}{2}\right) + \sigma D\left(\frac{T^3}{12} + \frac{T_o^4}{4T} - \frac{T_o^3}{3}\right)\right] \quad (S8b)$$

where $\Delta H_{f,Li}^{o}$ is the standard enthalpy of formation of Li metal. In Eq. S8b, the Shomate polynomial for LiC_6 is not known. However, experimental data for LiC_6, specifically its standard enthalpy of formation ($\Delta H_{f,LiC_6}^{o}$) and heat capacity C'_{P,LiC_6} are known. Thus, C'_{P,LiC_6} represents the integrated heat capacity data and is its own separate term in the heat capacity integration for the calculation of standard potential for LiC_6:

$$\int_{T_o}^{T} \Delta C_P \, dT = \int_{T_o}^{T} \left(C_{P,LiC_6}(T) - C_{P,Li}(T) - C_{P,C_6}(T)\right) dT$$

$$\int_{T_o}^{T} \Delta C_P \, dT = \underbrace{\int_{T_o}^{T} C_{P,LiC_6}(T) dT}_{C'_{P,LiC_6}} - \int_{T_o}^{T} \underbrace{\left(C_{P,Li}(T) + C_{P,C_6}(T)\right)}_{\sigma} dT$$

Thus, σB, σC, and σD represent the sum of heat capacity coefficients for solid Li metal and graphite (e.g., $\sigma B = B_{Li}^{solid} + B_{graphite}$).

From Ayache et al.[5], C'_{P,LiC_6} at temperatures between +30 °C and -40 °C was determined to be 5,249.85 J/mol/K. Similarly from Reynier et al.[6], $\Delta H_{f,LiC_6}^{o}$ was determined to be -12 kJ/mol.

The plots of Eq. S8a and Eq. S8b are thus shown in Figure S8.

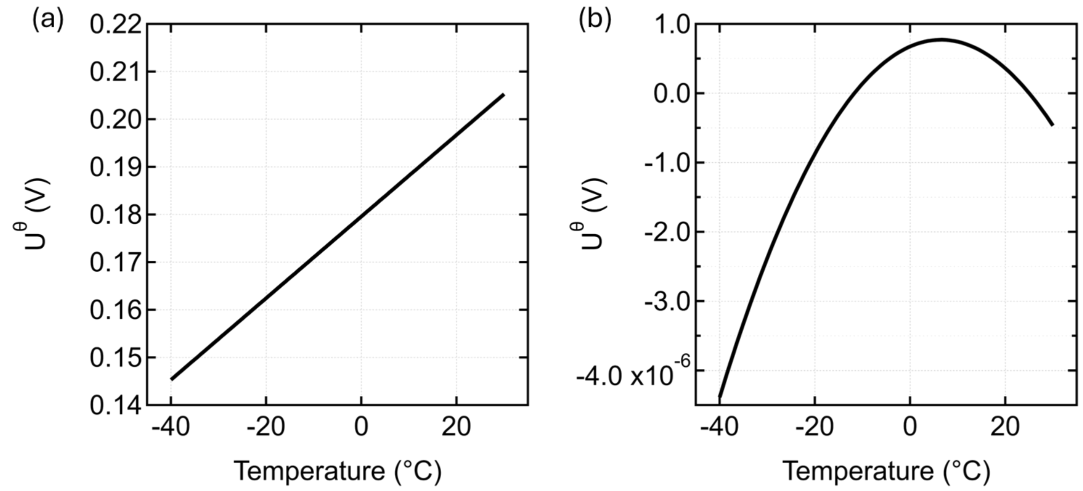

Figure S8. Result of calculating how the standard potential changes as a function of temperature for **(a)** Li^+ intercalation into graphite assuming a LiC_6 stage 1 formation, and **(b)** Li plating on graphite. The standard potentials (U^θ) are in reference to Li/Li^+.

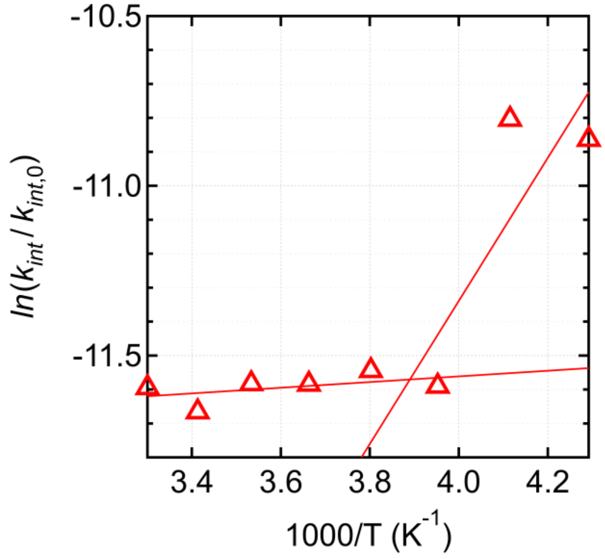

Figure S9. Arrhenius plot for the rate constant k_{int} if the time duration for Li^{+} intercalation (t_{int}) was defined at the start of the constant-current measurement up to the local cell potential minimum.

Supporting Text & Calculations

Text S3. Calculation of Mass-Transfer Limiting Current Through Electrolyte

The following calculation quantifies the limiting current density that would place the Li^+ cation reduction mechanism (Scheme 1, Main Text) under a mass transport-limited regime.

The limiting current can be calculated by the following (Eq. S9):

$$i_l = \frac{nF\mathcal{D}C_{Li^+}}{(1-t_+)\delta} \qquad (S9)$$

Where n is the number of electrons transfer for electrochemical redox, F is the Faraday's constant, $\mathcal{D}$ is the diffusion coefficient of Li^+ through the electrolyte, C_{Li^+} is the bulk concentration of Li^+ cations in the electrolyte, t_+ is the Li^+ transference number, and δ is the boundary layer thickness.

The following values were used (Table S3):

Table S3. Values used for limiting current calculation through electrolyte

Parameter	Value	Reference
Electrons Transferred (n)	1	Constant
Faraday's Constant (F)	96485 C/mol	Constant
Li^+ Concentration (C_{Li^+})	1 mol/dm^3	Selected
Diffusion Coefficient of Li^+ through 1 M $LiPF_6$ in Propylene Carbonate ($\mathcal{D}_{Li^+}$)	4×10^{-6} cm^2/s	Nishida et al.[7]
Li^+ transference number (t_+)	0.4	Valøen and Reimers[8]
Boundary Layer Thickness (δ)	400 µm	Ota et al.[9]

The limiting current assuming mass transport-limitations through the electrolyte becomes:

$$i_l = \frac{96485\,\frac{A \cdot s}{mol} \times 4 \times 10^{-6}\,\frac{cm^2}{s}\left(\frac{1\,m^2}{10^4\,cm^2}\right) \times 1\,\frac{mol}{dm^3}\left(\frac{1\,dm^3}{10^{-3}\,m^3}\right)}{(1-0.4) \times 400 \times 10^{-6}\,m} \times \left(\frac{1000\,mA}{1\,A}\right)\left(\frac{1\,m^2}{10^4\,cm^2}\right)$$

$$i_l \approx 16.08\,\frac{mA}{cm^2}$$

Thus, the expected limiting current assuming mass transport-limitations through the electrolyte phase is greater than the current densities tested in this work (0.1 mA/cm^2 to 10 mA/cm^2).

References

1. Dahn, J.R. (1991). Phase Diagram of Li_xC_6. Physical Review B *44*, 9170-9177. 10.1103/physrevb.44.9170.
2. Dahn, J.R., Fong, R., and Spoon, M.J. (1990). Suppression of staging in lithium-intercalated carbon by disorder in the host. Physical Review B *42*, 6424-6432. 10.1103/physrevb.42.6424.
3. Fuller, T.F., and Harb, J.N. (2018). Electrochemical Engineering (Wiley).
4. Domalski, E.S., and Hearing, E.D. (2023). NIST Chemistry WebBook. 10.18434/T4D303.
5. Ayache, C., Bonjour, E., Lagnier, R., and Fischer, J.E. (1980). Specific heat of LiC6 from 4-300 K. Physica B+C *99*, 547-550. https://doi.org/10.1016/0378-4363(80)90294-6.
6. Reynier, Y., Yazami, R., and Fultz, B. (2003). The entropy and enthalpy of lithium intercalation into graphite. J. Power Sources *119-121*, 850-855. 10.1016/s0378-7753(03)00285-4.
7. Nishida, T., Nishikawa, K., and Fukunaka, Y. (2008). Diffusivity Measurement of LiPF6, LiTFSI, LiBF4 in PC. ECS Transactions *6*, 1. 10.1149/1.2831921.
8. Valøen, L.O., and Reimers, J.N. (2005). Transport Properties of LiPF6-Based Li-Ion Battery Electrolytes. J. Electrochem. Soc. *152*, A882. 10.1149/1.1872737.
9. Ota, M., Izuo, S., Nishikawa, K., Fukunaka, Y., Kusaka, E., Ishii, R., and Selman, J.R. (2003). Measurement of concentration boundary layer thickness development during lithium electrodeposition onto a lithium metal cathode in propylene carbonate. Journal of Electroanalytical Chemistry *559*, 175-183. https://doi.org/10.1016/j.jelechem.2003.08.020.
